# The oral microbiome is associated with HPA axis response to a psychosocial stressor

**DOI:** 10.1038/s41598-024-66796-2

**Published:** 2024-07-09

**Authors:** Eleftheria G. Charalambous, Sophie B. Mériaux, Pauline Guebels, Claude P. Muller, Fleur A. D. Leenen, Martha M. C. Elwenspoek, Ines Thiele, Johannes Hertel, Jonathan D. Turner

**Affiliations:** 1 Immune Endocrine Epigenetics Research Group, Department of Infection and Immunity, Luxembourg Institute of Health, 29, rue Henri Koch, 4354 Esch-sur-Alzette, Luxembourg; 2 Faculty of Science, Technology and Medicine, University of Luxembourg, Esch-sur Alzette, Luxembourg; 3 Department of Psychiatry and Psychotherapy, University Medicine Greifswald, Greisfwald, Germany; 4 Department of Psychology, University of Cyprus, 2109 Nicosia, Cyprus; 5 School of Medicine, National University of Ireland, Galway, Ireland; 6 Ryan Institute, National University of Galway, Galway, Ireland; 7 Division of Microbiology, National University of Galway, Galway, Ireland; 8 APC Microbiome Ireland, Cork, Ireland; 9 German Center for Cardiovascular Diseases (DZHK), Partner Site Greifswald, Greifswald, Germany

**Keywords:** Early life adversity, Microbiome, Developmental origins of health and disease, Stress, HPA axis, Host-microbiome interactions, Microbial communities, Biomarkers, Neuroimmunology, Social neuroscience, Stress and resilience

## Abstract

Intense psychosocial stress during early life has a detrimental effect on health-disease balance in later life. Simultaneously, despite its sensitivity to stress, the developing microbiome contributes to long-term health. Following stress exposure, HPA-axis activation regulates the “fight or flight” response with the release of glucose and cortisol. Here, we investigated the interaction between the oral microbiome and the stress response. We used a cohort of 115 adults, mean age 24, who either experienced institutionalisation and adoption (n = 40) or were non-adopted controls (n = 75). Glucose and cortisol measurements were taken from participants following an extended socially evaluated cold pressor test (seCPT) at multiple time points. The cohort´s oral microbiome was profiled via 16S-V4 sequencing on microbial DNA from saliva and buccal samples. Using mixed-effect linear regressions, we identified 12 *genera* that exhibited an interaction with host’s cortisol-glucose response to stress, strongly influencing intensity and clearance of cortisol and glucose following stress exposure. Particularly, the identified taxa influenced the glucose and cortisol release profiles and kinetics following seCPT exposure. In conclusion, our study provided evidence for the oral microbiome modifying the effect of stress on the HPA-axis and human metabolism, as shown in glucose-cortisol time series data.

## Introduction

The physiological stress response is the body’s natural reaction to external stressors that causes physical, psychological or emotional strain. The response to a psychosocial stressor is highly individual, and dependent on many environmental factors such as prior stress exposure and potentially the microbiome^1^. While the physiological response to stressors is part of everyday life, there are periods of life during which we are particularly affected. Stressful events occurring very early in life are particularly harmful, and are intimately linked to health-disease balance in later life^2,3^. In the mid-1980s, Barker and Osmond introduced the developmental origins of health and disease (DOHaD), where the environment in the first 1000 days was hypothesised to shapes health and disease profiles lifelong^4^. Although Barker and Osmond were initially interested in foetal nutrition, this has now expanded to cover almost all negative experiences in this 1000-day period, and has led to interest in early-life adversity (ELA). ELA is a rather diffuse concept, covering many different forms of potentially adverse environmental exposure within the first 1000 days. Many studies have subsequently investigated the molecular mechanisms linking ELA to psychobiological, behavioural, immunological and disease phenotypes^5–15^.

Upon exposure to a stressor, the autonomic nervous system and the hypothalamus pituitary adrenal (HPA) axis activates and coordinates the “fight or flight” response via the release of catecholamines and glucocorticoids^5,16,17^. In parallel, glucose is produced and released^16^. The current dogma is that this is a glucocorticoid-mediated process. Exposure to ELA has lifelong effects on HPA axis regulation and glucocorticoid levels, consequently dysregulating glucose release and metabolism dynamics^5,8^. However, it remains unclear exactly how these stress and metabolic processes are linked and regulate one another. Such psychochosocial stressors are readily modelled using paradigms such as the Trier Social Stress Test^18^ or the socially evaluated cold pressor test (seCPT)^19^ where the HPA axis is readily activated.

**Figure 1:**
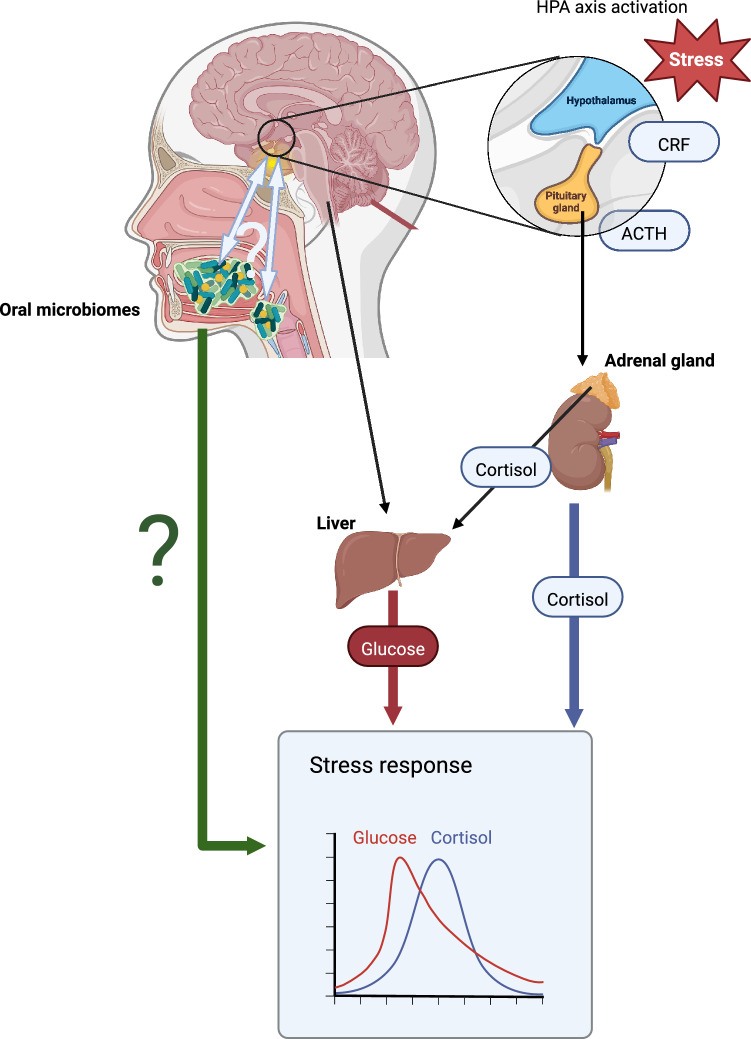
Outline of our study hypothesis. Psychosocial stressors activate the HPA axis with release of CRF and ACTH leading to the adrenal gland releasing cortisol as part of the stress response (lowerbox). At the same time Cortisol induces a glucose response from the liver. There is then a crosstalk between the oral microbiome and central stress processes. Our question is how the oral microbiome interacts with the overall cortisol and glucose response to a psychosocial stressor.

The microbiome, seeded at birth, is also known to have a pivotal role on the establishment of an individual’s long-term health trajectory. Furthermore, both the oral and gastro-intestinal microbiomes are shaped by ELA^9–15,20,21^. There has been a recent increase in interest in the oral microbiome (OM). After exposure to ELA, dysbiosis of the OM, with increased abundance of pathogenic taxa, leads to poor oral health. Poor oral health is part of the pathophysiological presentation of many ELA-associated diseases, including cardiometabolic, mental, autoimmune and allergic diseases. Furthermore, the paradigm of gut – brain – axis and oral – brain – axis is now more widely-studied, strengthening the evidence of a constant communication between the gut and oral microbial communities with their hosts^17,22^. Interestingly, the OM is not only sensitive to ELA, but is also sensitive to both cortisol and glucose that may be dysregulated by ELA^17,23,24^. This raises the interesting hypothesis that dysbiosis in the microbiome interacts with the stress response. Moreover, this may represent a mechanism by which ELA alters the stress response (Fig. 1).

Here, we investigated the interplay between the OM and the psychosocial stress response. To do this, we take advantage of the variability in both the stress response and the two distinct OM (buccal and salivary communities) within our EpiPath cohort of ELA-exposed individuals and controls. Here, both the acute stress response and the OM were reshaped by early life psychosocial stress exposure^5,8,20^. The use of such a “natural experiment” that exaggerates inter-individual differences allowed us to gain a clear insight into the role of the OM in determining the host’s stress reaction after HPA axis activation using the seCPT.

## Results

As previously reported, glucose and cortisol levels are raised by the seCPT in the EpiPath cohort^5^. The exposure to institutionalisation-adoption made subtle but statistically significant changes to the kinetic profile of the stress induced release of cortisol and glucose. Briefly, we saw a rise in glucose levels from 101.8 + /- 12.6 mg/dL to 136 + /- 21.7 mg/dL and cortisol rose from 0.27 + /- 0.14 µg/dL to 0.55 + /- 0.29 µg/dL (Supplementary Table 1). Here, we took the two arms of the EpiPath cohort together, using the exposure to ELA as a source or variance in metabolic and hormonal responses to stress coupled with differences in the microbiome. In the combined arms of the cohort, both cortisol and glucose showed a clear stress-induced rise that eventually falls to time-appropriate background level (Supplementary Fig. 1A B and C). Analysing the cortisol and glucose time series via linear mixed effect regression models, we identified a total of 12 *taxa*, 10 that were present in the buccal and 2 in the salivary microbiome prior to the stress test, that subsequently interacted with either the cortisol or the glucose response to the seCPT. In addition, diversity and evenness metrics, Inverse Simpson index and Shannoneven index respectively interacted with both glucose and cortisol kinetics. Significant interactions are summarised in Table 1, and complete data are in Supplementary Tables S1- S8.

**Table 1:** Results Summary.

Taxa	Buccal microbiome	Salivary microbiome	Interaction analysis
Absconditabacteriales	Cortisol	–	Presence/absence
	Glucose	–	Presence/absence
Acinetobacter	Cortisol	–	Presence/absence
Clostridia UCG14	Cortisol	–	Presence/absence
Campylobacter	–	Cortisol	Presence/absence
Cardiobacterium	–	Cortisol	Presence/absence
Oxalobacteraceae	Glucose	–	Presence/absence
Sphingomonas	Glucose	–	Presence/absence
	Glucose	–	Abundance
Bradyrhizobium	Glucose	–	Abundance
Comamonadaceae	Glucose	–	Abundance
Flavobacterium	Glucose	–	Abundance
Methylobacterium -Methylobrubrum	Glucose	–	Abundance
Paucibacter	Glucose	–	Abundance

Diversity Indices			
Inverse Simpson Diversity Index	Cortisol	–	–
	Glucose	–	–
Shannon Evenness Index	Glucose	Cortisol	–

### Glucose and cortisol kinetics associate with diversity and evenness indices

Following the clear indications that the taxonomic composition leads to a modified cortisol and glucose response to the seCPT, we hypothesized that diversity of the microbial composition also can modify the biological response to stress. Similar to the abundance analyses above, we used diversity indices scores from Inverse-Simpson diversity index and Shannon evenness index as dimensional interaction terms together with plasma glucose and saliva cortisol levels. Interestingly, diversity and evenness of the buccal microbiome showed an interaction with glucose kinetics while salivary microbiome showed no interactivity (Supplementary Table 8A). To visualize the dimensional interaction terms, diversity and evenness scores were stratified according to tertiles. Increased Inverse-Simpson score (> 66 th percentile) was associated with delayed and lower glucose peak (Supplementary Fig. 2B, LR-test: p = 168E-02). Likewise, increased Shannon evenness score was also associated with delayed and lower glucose peak (Supplementary Fig. 2D, LR-test: p = 447E-04). Notably lower score (< 33th percentile) in both diversity and evenness showed the highest glucose peak while for median (33 th-66 th percentile) diversity scores glucose peak was lower (Supplementary Fig. 2B, LR-test: p = 1,68E-02, Supplementary Fig. 2D, LR-test: p = 4,47E-04). Intriguingly, only evenness score of the salivary composition and diversity score of the buccal composition exhibited an interaction with cortisol kinetics (Supplementary Table 8B). For the buccal microbiome, increased Inverse-Simpson score was associated delayed cortisol clearance (Supplementary Fig. 2F, LR-test: p = 119E-02). For the salivary microbiome, lower Shannon evenness score (< 33 th percentile) was associated delayed cortisol clearance (Supplementary Fig. 2F, LR-test: p = 3,33E-03).

### Cortisol response depends on the oral taxonomic profile

**Figure 2:**
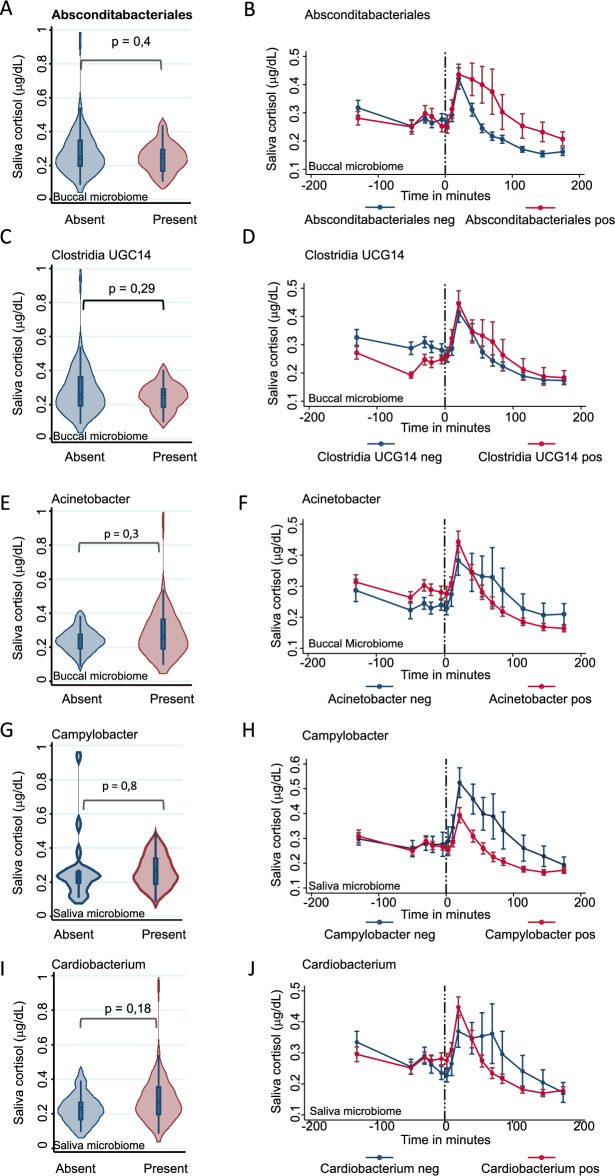
Cortisol response depends on the oral taxonomic profile FDR corrected significant. taxa with differential cortisol kinetics depending on presence absence of buccal and salivary microbiome Left panel (**A**, **C**, **E**, **G**, **J**). Violin plots for baseline cortisol measurement at 5 min before seCPT p values from linear regressions adjusted for age, sex, BMI and study group Right panel (**B**, **D**, **F**, **H**, **I**) time series of cortisol measurements in saliva with standard errors as the error bars.

Here, we describe the results regarding the cortisol response. Our results revealed three taxa from the buccal community interacting with the salivary cortisol response to stress in the buccal community (Fig. 2, Supplementary Tables 2 and 3). The presence of *Absconditabacteriales*, *Clostridia UGC14* and *Acinetobacter* had no significant effect on the baseline levels of cortisol (Fig. 2A,C,E *Absconditabacteriales*: β = -0.03, 95%-confidence interval (95%-CI): − 0.10 –0.04, p = 0.4; *Clostridia UGC14*: β = -− 0.04, 95%-CI: − 0.10–0.03, p = 0.29; *Acinetobacter:* β = 0.04, 95%-CI: -0.03–0.10, p = 0.3). Nevertheless, the presence of Absconditabacteriales and *Clostridia UGC14* was associated with different cortisol biochemical dynamics, displaying a prolonged clearance of cortisol following stress (Fig. 2B,D *Absconditabacteriales*: likelihood ratio (LR) test: p = 1.89E-07, FDR < 0.05, *Clostridia UGC14*: LR-test: p = 3.34E-03, FDR < 0.05). In contrast, *Acinetobacter* presence, was associated with accelerated clearance of cortisol (Fig. 2F *Acinetobacter:* LR-test: p = 2.61E-03, FDR < 0.05). In addition, analyses of salivary communities showed two *genera* interacting with cortisol levels in saliva after the CP test, while none of the genera had an effect on baseline cortisol levels after correction for multiple testing (see Supplementary Tables 2 and 3). Neither *Campylobacter* nor *Cardiobacterium* presence was associated with baseline cortisol (Fig. 2G *Campylobacter*: β = -0.01, 95%-CI: -0.08—0.06, p = 0.8; Fig. 2J *Cardiobacterium*: β = 0.05, 95%-CI: − 0.02–0.12, p = 0.18). Yet, the presence of *Campylobacter* associated with a lower cortisol reaction to CP in general (Fig. 2H *Campylobacter*, LR-test: p = 3.43E-06, FDR < 0.05). Similarly, *Cardiobacterium’s* presence associated with a higher cortisol peak and faster clearance (Fig. 2I *Cardiobacterium*, LR-test: p = 3.96E-04, FDR < 0.05). Detailed summary statistics for both cortisol baseline levels and the overall time series are available in Supplementary Tables 2 and 3 respectively. In conclusion, community composition both in the saliva as in the buccal microbiome interacted with the measured cortisol response to CP.

### Glucose response depends on the oral taxonomic profile

**Figure 3:**
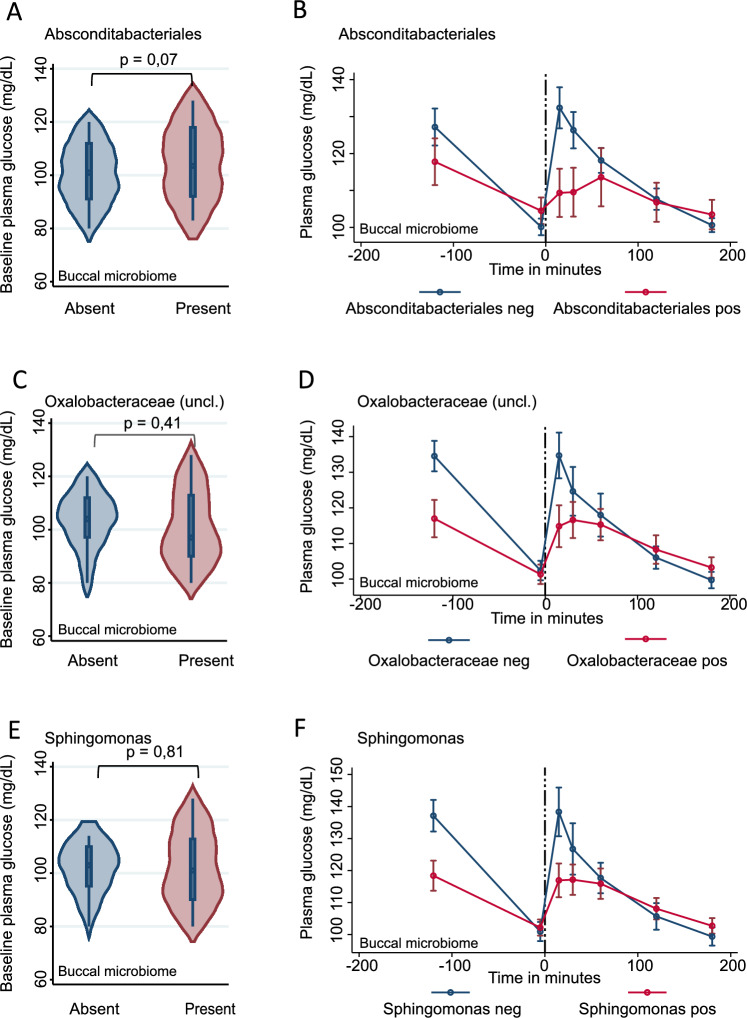
Glucose response depends on the oral taxonomic profile FDR corrected significant taxa with differential plasma glucose kinetics depending on presence absence of buccal microbiome Left panel (**C**, **E**). Violin plots for baseline cortisol measurement at 5 min before seCPT, p values from linear regressions adjusted for age, sex, BMI and study group Right panel (**D**, **F**) time series of plasma glucose measurements with standard errors as the error bars.

Next, we explored the relation between the glucose response to the seCPT and oral microbiome. To this end, we conducted linear mixed regression modelling analyses in an analogous way as described above, utilizing however, the plasma glucose levels as response variable instead of the saliva cortisol concentrations (Fig. 3, Supplementary Tables 4 and 5). While the salivary microbiome showed no interactions, three taxa from the buccal community appeared to interact with glucose stress response despite showing no significant association with the baseline plasma glucose levels 5min before stress test (Fig. 3A *Absconditabacteriales*: β = 7.61, 95%-CI: -0.73 — 15.96, p = 0.07; Fig. 3C *Oxalobacteraceae*: β = 3.16, 95%-CI: -4.55 — 10.87, p = 0.41; Fig. 3E *Sphingomonas*: β = 0.97, 95%-CI: -7.13 — 9.06, p = 0.81). In contrast to the baseline levels, the presence of *Absconditabacteriales* in the buccal microbiome was associated with muted plasma glucose response, while participants without *Absconditabacteriales* showed a clear stress-induced glucose response (Fig. 3B *Absconditabacteriales,* LR-test: p = 1.62E-04, FDR < 0.05). Moreover, *Oxalobacteraceae* and *Sphingomonas* were associated with a reduced glucose response globally (Fig. 3C *Oxalobacteraceae,* LR-test: p = 3.10E-03, FDR < 0.05; Fig. 3F *Sphingomonas,* LR-test: p = 3.49E-03, FDR < 0.05,). Notably, both species co-occurred with each other in the analyzed buccal microbiomes, showing therefore parallel association patterns with plasma glucose levels (Fig. 3 C, E). Detailed summary statistics for glucose baseline levels available at the Supplementary Table 4 and for glucose time series at Supplementary Table 4.

### Glucose clearance associates with a higher taxonomic abundance

**Figure 4:**
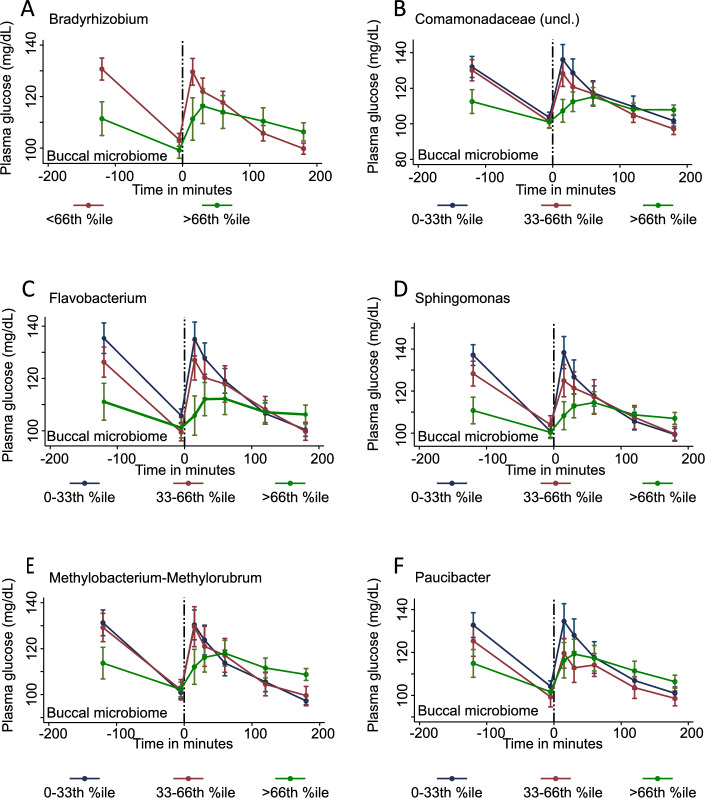
Glucose clearance associates on the oral taxonomic profile FDR corrected significant taxa with differential plasma glucose kinetics depending on taxonomic abundance of buccal microbiome Time series of plasma glucose measurements with standard errors as the error bars depending on the taxonomic abundance of (**A**) *Bradyrhizobium* (**B**) *Comamonadaceae* (**C**) *Flavobacterium* (**D**) *Methylobacterium Methylorubrum* (**E**) *Sphingomonas* (**F**) *Paucibacter*.

While in the analyses above, we worked with dichotomized genus variables, in the next string of linear mixed regression models, we utilised dimensional interaction terms *between the relative abundance values and the levels of plasma glucose and salivary cortisol, respectively*. Neither the buccal, nor the saliva microbiome showed any significant interactions on the abundance level after correction for multiple testing with the saliva cortisol levels. However, for six genera in the buccal microbiome, the abundance was associated with modificiations of the glucose response (Fig. 4, Supplementary Table 6).

For visualization of the dimensional interaction terms, abundances were stratified in according to tertiles. High *Bradyrhizobium* abundance (> 66th percentile) was associated with delayed and lower peak, while showing longer clearance in tendency (Fig. 4A *Bradyrhizobium*, LR-test: p = 9.25E-04 , FDR < 0.05). Abundances of *Commamonadaceae*, *Flavobacterium* and *Sphingomonas* showed a similar interaction where lower abundances 0-33th percentile and 33th—66th percentile had a similar response, whereas individuals with abundance of these taxa > 66th percentile exhbited a muted glucose response with a less clear peak (Fig. 4B *Commamonadaceae*, LR-test: p = 1.42E-04, FDR < 0.05; Fig. 4C *Flavobacterium*, LR-test: p = 5.99E-03, FDR < 0.05; Fig. 4D *Sphingomonas*, LR-test: p = 2.53E-04, FDR < 0.05). Furthermore, 0-33th percentile and 33th—66th percentile abundances of *Methylobacterium-Methylorubrum* showed a very similar response while the individuals with abundance higher than 66th percentile demonstrated a lesser glucose response with longer clearance (Fig. 4E *Methylobacterium-Methylorubrum*, LR-test: p = 3.90E-03, FDR < 0.05). Lastly, *Paucibacter* abundance showed a different interaction with the glucose stress response. Individuals with less than 33th percentile of *Paucibacter* showed a clear glucose response to CP, while individuals with abundances in the two lower tertiles had a lower glucose response (Fig. 4E *Paucibacter*, LR-test: p = 4.13E-03, FDR < 0.05).

## Discussion

In this study, we provided clear evidence for a link between the composition of the oral microbiome and both the hormonal and metabolic response to a laboratory psychosocial stressor. Our EpiPath cohort that were exposed to early-life adversity 20 years earlier had both a reshaped stress responses and microbiota, providing variance in both to enable us to identify interactions between bacterial taxa and the stress response. Within this paradigm, the stress-induced cortisol response was associated with 5 bacterial taxa originating from either the buccal or salivary microbiomes. Furthermore, 8 taxa from buccal microbiome when present, or present in increased abundance interacted with the stress induced glucose response, determining the kinetics of glucose following seCPT exposure. Fascinatingly we also showed that it is not only the presence and abundance of particular taxa that interfere with the HPA-axis stress response but also the community as a unit in terms of diversity and evenness. In particular, in our study we observed an interaction of plasma glucose kinetics with both diversity and evenness of buccal composition. In contrast, salivary cortisol kinetics were found to be associated with only diversity of buccal composition whilst also associated with evenness of salivary composition. This highlights that individual taxa are equally important in host’s physiology and the dynamics of the microbial community. Overall our data demonstrate a clear interaction between the oral microbiome and both the hormonal and metabolic response to a psychosocial stressor. This builds on our previous report that buccal and salivary microbiome compositions are different, even in healthy controls^20^.

Many studies have linked ELA to a dysregulated HPA axis stress response^25–27^. Concurrently, numerous studies investigating gut-brain axis and/or oral-brain axis have reported that microbiota respond to host hormones resulting to change in bacterial gene expression^17,28–33^. In particular, evidence from the oral microbiome suggests that exposure to high cortisol levels results to upregulation of virulence factors (lipopolysaccharide, fimbriae or gingipains) in periodontitis associated taxa, *Fusobacterium* and *Poprhymonas* resulting to a global shift of the composition towards a pathogenic type^17,28,29,34,35^. This ability of the OM to react to the host’s hormonal response is part due to resilience mechanisms determining the community composition and driving homeostasis^25,36^. During the HPA activation and stress response, in addition to cortisol, catecholamines are also released. In vitro studies have shown that these can promote or inhibit the growth of OM taxa known to be associated with periodontitis and evidently lead to stress induced taxonomic shifts in the OM composition^25,29,35,37^. In a similar manner, our data suggest a clear interaction between the host’s stress hormones and the composition of the microbiome. However, the directionality of the interaction remains unclear. It is possible that the host’s stress response is modified by the microbiome. On the other hand, 24 years after institutionalisation, it is more probable that, as in vitro, the microbiome has established a new homeostasis based on the ELA-specific stress and metabolic profile of the individual, with stress hormones and glucose promoting or inhibiting growth of specific taxa.

Glucose can be a substrate in pentose phosphate pathways for certain taxa to generate other energy sources. Other taxa such as *Sphingomonas* produce glucose through other metabolic pathways as their final energy source^38–41^. Additionally, in vitro studies suggest that glucose concentration affects bacterial mobility and more precisely the swimming – swarming behaviour^42–46^. Swarming is often observed in stress or disease state^42^. This is believed to occur through a glucose-dependent quorum sensing mechanism^41,45,47^. This process requires different carbon sources for carbon catabolite repression (CCR) which is broadly believed to be glucose^41,48^. Additional in vitro data suggest that glucose is the most accessible and preferred carbon source, driving faster bacterial growth^40,47^. CCR dependent quorum sensing contributes to an increase in virulence proteins^41,48–50^. In a similar manner, there is also preliminary evidence that several taxa within the human microbiome can also metabolise and use cortisol as a substrate^51^.

In this complex system where ELA and stressful events can lead to dysbiosis and a ruffled balance of health-disease the light falls in the microbiome in order to understand underlying mechanisms of action. The microbiome and more importantly the oral communities have been showed to be involved in the modulation of neurological process, to shape behaviour and even cognition by interacting with the neuroendocrine system^17^. This modulation may, in part, be due to metabolites or other small molecules secreted by the microbiome and absorbed by the host. On the other hand, as saliva contains molecules with antimicrobial properties, the OM has also evolved to recognize and respond to such signals^23,24,52–54^. This re-enforces the idea that there is an equilibrium or homeostasis between the host’s stress response and the OM composition.

The OM possess a set of sociomicrobiological skills, scientifically known as quorum sensing (QS), a form of intra- and inter- species communication that allows the OM to sense and modulate the host environment^17,55,56^. QS mechanisms are the basis of how bacteria react and regulate stress that can ensure survival and homeostasis of a functional bacterial community^17,57^. Both cortisol and glucose are known to interact with QS whilst individual taxa are able to counteract by releasing particular autoinducer peptides, which often enhances their virulence and growth-speed^56,58,59^. Furthermore, some microbial metabolites can neutralise the action of the QS peptides aiming to promote homeostasis within the community, such metabolites are D-Galactose and D-arabinose^60^. These properties of the OM have ensured long-term stability, resilience and robustness for its communities^61,62^. Furthermore, it is the key on how oral taxa can drive oral inflammation and impact systemic health later on^63^. Overall these pre-existing evidence explain the observations of our study and strengthen our hypothesis that the OM interferes with HPA axis activation and glucose-cortisol stress response. Further mechanistic-focus studies are essential to explore exactly how each of these QS mechanisms and bacterial metabolic properties relate to this OM-HPA interaction. As such, it would be interesting in future studies to measure salivary QS molecules to examine their role in determining the microbiome stress interaction.

Gut-brain axis research provides indications of a bidirectional interaction between microbiome and HPA axis^64^. Microbial metabolites. Microbial metabolites contribute to downstream signaling and activation of the HPA axis stress response and release of corticosterones. Whilst a chronically activated HPA axis results to microbiome-related chronic inflammatory diseases such as irritable bowel syndrome (IBS) it also contributes to the pathogenesis and progression of e.g. diabetes, depression and neurodegenerative diseases^65–72^. In addition, long-term stress linked to impacted intestinal permeability and changes on the microbiome and concequently interfere with psychiatric phenotypes and medication^65–69^, leading us to conjecture that the oral microbiome is equally capable of promoting such interactions.

Our observation that host’s stress response can be altered based on the composition of the oral microbiome brings opportunities for future research. Collection of oral swabs is widely performed in many research fields including psychobiology, lifestyle and other social to clinical research areas. Such samples are consisting a non-invasive, cost-effective and accurate diagnostic approach that is optimisable into personalized medicine strategies^73,74^. The plausible use of such samples can enlighten investigations on the interaction of host-microbe and the role of the microbiome in oral and systemic health.

Our study, like all others, has limitations. First, our study is observational in design, such that we were not able to identify the direction of the interaction between the stress reaction and changes in the microbiome. As such, the causal direction potentially underlying the effect modifications cannot be determined. However, our data can serve for informing later targeted mechanistic studies. Additionally, the glucose and cortisol measurements from the EpiPath cohort were only measured for a smaller set of participants based on the availability of the biosamples. Our EpiPath cohort was primarily conceived as an ELA study, and while its sample size is considerable for such a study. Nonetheless, such a sample size is considered to be small for a microbiome study, especially and given the inter-personal variability in stress-response, meaning that we were not able to model the effects of ELA on the microbiome-stress interaction. The small sample size also limited our possibilities to investigate rare taxa with high zero-inflation. Moreover, the limited quantity of saliva meant that 16S amplicon sequencing was used to ensure good-quality data, leading to limited taxonomic resolution, although this is counterbalance by the reduced risk of missing a particular genus^20^. In addition, due to the original scientific question that the EpiPath was conducted for, microbiome-specific metadata such as, diet habits and oral health status were not collected. Knowing that some of the taxa we identified to interact with the stress response including *Acinetobacter*, *Campylobacter* and *Sphingomonas,* are associated with periodontitis and other systemic diseases^67,71,72,75–80^, information on the participants’ oral health would have strengthen the mechanistic potential of our dataset.

## Conclusion

Our data show a clear alteration of the host’s stress response as evidenced through the analyses of repeated glucose and cortisol measurements in relation to the taxonomic and structural composition of the oral microbiome. We have, for the first time, clearly shown from this observational study that there is a clear interaction between taxa from the oral microbiome and the host’s stress response. In the case of our EpiPath ELA cohort, it is most probable that a dysregulated endocrine stress reaction is able to cause dysbiosis of the OM. This interaction between the two systems may play a role in re-establishing homeostasis of the OM following a stress trigger.

## Materials and methods

### Participants and bacterial abundance data

In this study we used previously published buccal and salivary microbiome abundance data from sequencing of the V4 region of the 16S gene in our EpiPath cohort^20^. EpiPath is a cohort of post-institutionalisation adults and immediate social circle control participants that were brought up by their biological parents^6,8,81,82^. Microbial abundance data were from buccal swabs and salimetrics oral swabs taken upon arrival at the clinical centre. A subset of the EpiPath cohort underwent a socially evaluated cold pressor test (seCPT;^8^), and were used in this study. These participants did not differen in any characteristic measured from the rest of the cohort^8^ (Supplementary Table 1B). Median age at adoption was 4.3 months (IQR 0–15 months)^81^. EpiPath was approved by the Luxembourg National Research Ethics Committee (CNER, No 201303/10 v1.4) as well as the University of Luxembourg Ethics Review Panel (ERP, No 13–002). All participants provided written informed consent in compliance with the Declaration of Helsinki. All study participants received a small financial compensation for their time and inconvenience.

*Stress test:* As previously reported, a subset of the cohort underwent an extended seCPT^8^. During the seCPT they were asked to place both feet into 2–3°C water for 3min while performing a mental arithmetic task^83^. Blood and saliva were collected using EDTA coated tubes and Salimetrics Oral Swabs respectively at − 120 min, − 5 min, + 3 min (stress cessation), and then at 15, 30, 60, 120 and 180 min relative to T = 0 when the participant placed their feet in the water. EDTA tubes were centrifuged at 4°C for 15 min and plasma collected. Samples were stored at − 80°C prior to utilisation.

### Cortisol and glucose measurements

Salivary cortisol data was available from 70 participants, measured using the Salimetrics Salivary Cortisol ELISA kit (CV: 7% intra-assay, 11% inter-assay, Salimetrics, Cambridgeshire, UK). Plasma glucose was measured from the subset of 42 participants that had fully completed the seCPT^8^. The reduced number of participants for which glucose data was available was limited by the availability of the complete seCPT time-series plasma samples. These were briefly vortexed and placed on a fresh Accu-Chek strip (Accu-Chek, Roche) to quantify the plasma glucose concentration as described by Seal et al.^5^.

### Statistical analyses

For descriptive statistics, metric variables were expressed as means + /- standard deviations, categorical variables were expressed via proportions. A sample description can be found in Supplementary Table 1 For interaction analyses of the response to the CP with the oral microbiome, we utilised two types of analyses. First, for genera being present in 25–75% of all samples, we dichotomized the abundance of those genera (genus present vs. genus absent). Then, for each of these genera we generated linear mixed models for plasma glucose as well as salivary cortisol levels as the response variable. These mixed linear regression models included the age, sex, body mass index, and the time of measurement as fixed effect covariates and the individual as random effect variable. For plasma glucose, measurements from eight seCPT time points: − 120 min, − 5 min, + 3 min (stress cessation), and then at 15, 30, 60, 120 and 180 min relative to the onset of stress (T = 0) were available for each individual. Saliva cortisol measurements were available for a larger time-range -130, − 50, − 30 , − 20, − 5, 3, 10, 20, 40, 55, 70, 85, 11, 145, 175 min relative to the onset of stress (T = 0). The time variable was treated in minutes with the onset of CP test being set to zero. Importantly, the time point of measurement was treated as a categorical variable to allow for the expected non-linear response over time to the CP test. We then introduced interaction terms between the categorical time point variable and the dichotomized species presence and tested the model including the interaction terms against a model including all named covariates plus the dichotomized genus abundance through likelihood ratio tests. The likelihood ratio tests effectively test, whether the saliva cortisol, respectively plasma glucose, response is the same for individuals having a certain genus vs. not having a certain genus in their oral microbiome. This string of mixed linear regression models was performed for both the oral and the buccal microbiome.

Second, for genera being present in more than 50% of the samples, we performed analogous interaction analyses, utilising however the metric abundance variable instead of the dichotomized variable. Once again, significance was determined by likelihood ratio test of the model including covariates, main effects and time-point genus abundance interactions terms vs. the model only including covariates and main effects. Finally, we analysed metrics of microbial diversity for both compartments in analogous linear mixed effect regressions, determining significant time-diversity interactions on the glucose and cortisol response as before by likelihood ratio tests as before.

All reported p-values are two-tailed. Statistical analyses was performed with STATA 16/MP and the mixed models were performed using the “xtreg” command with the option “mle” to specify maximum likelihood estimation. We corrected for multiple testing using the false discovery rate (FDR) and an FDR < 0.05 was considered to be significant. Where available, effect sizes (β) are reported. Summary statistics of the performed association analyses can be found in Supplementary Tables S1–S6.

### Supplementary Information

Supplementary Information.

## Data Availability

All data are available from the corresponding author (JDT) upon reasonable request under the condition that a suitable EU General Data Protection Regulation – compliant data sharing and processing agreement can be reached.
