# Supplementary material for: The oral microbiome is associated with HPA axis response to a psychosocial stressor
Source: Sci Rep. 2024 Jul 9;14:15841. doi: 10.1038/s41598-024-66796-2 (PMC11233668; doi:10.1038/s41598-024-66796-2)
Supplement: Supplementary file 1 — Supplementary Information. [file 41598_2024_66796_MOESM1_ESM.pdf]

Supplementary Figure 1

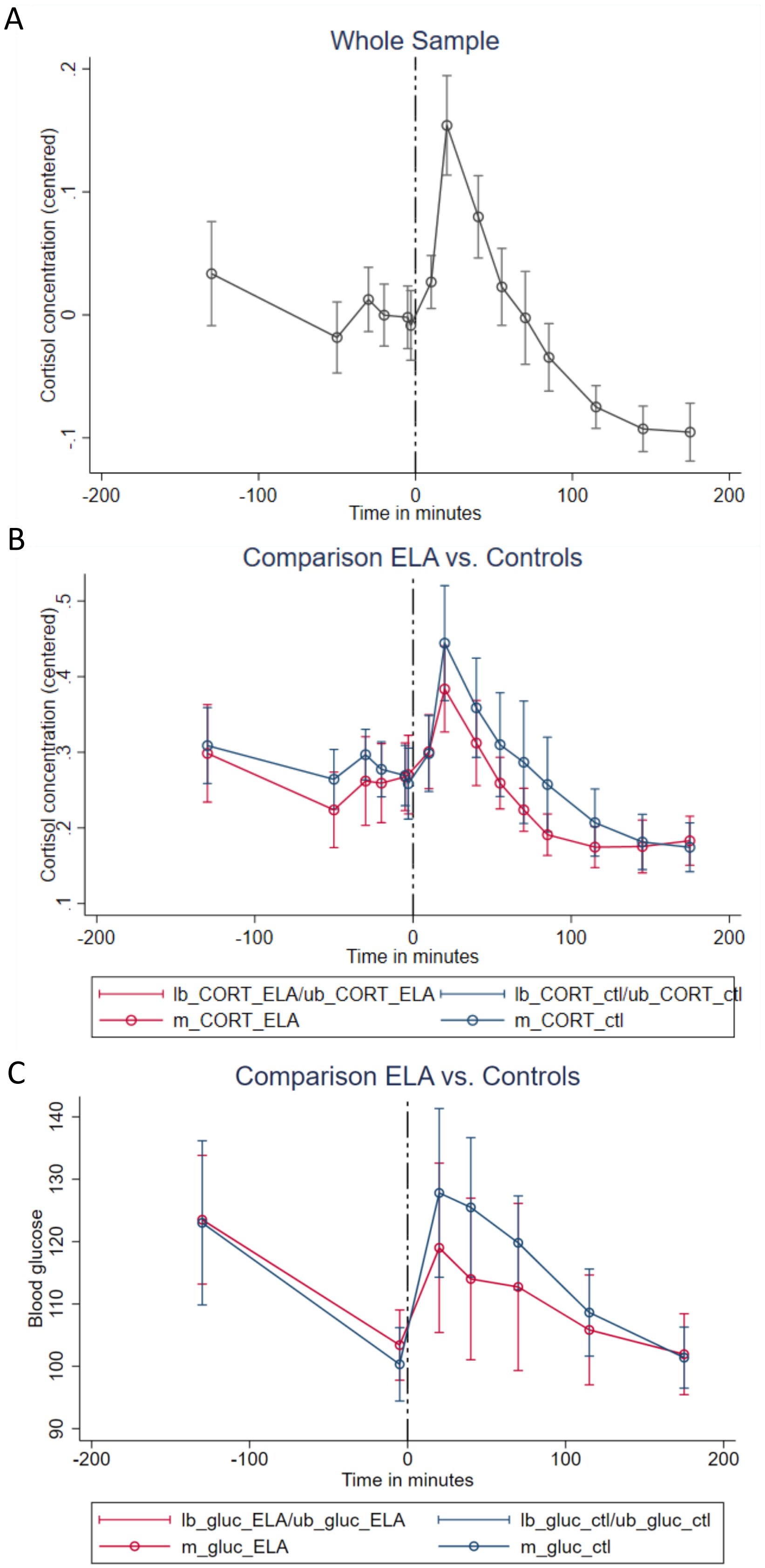

Supplementary Figure 2: Diversity Indices associated with glucose/cortisol stress kinetics

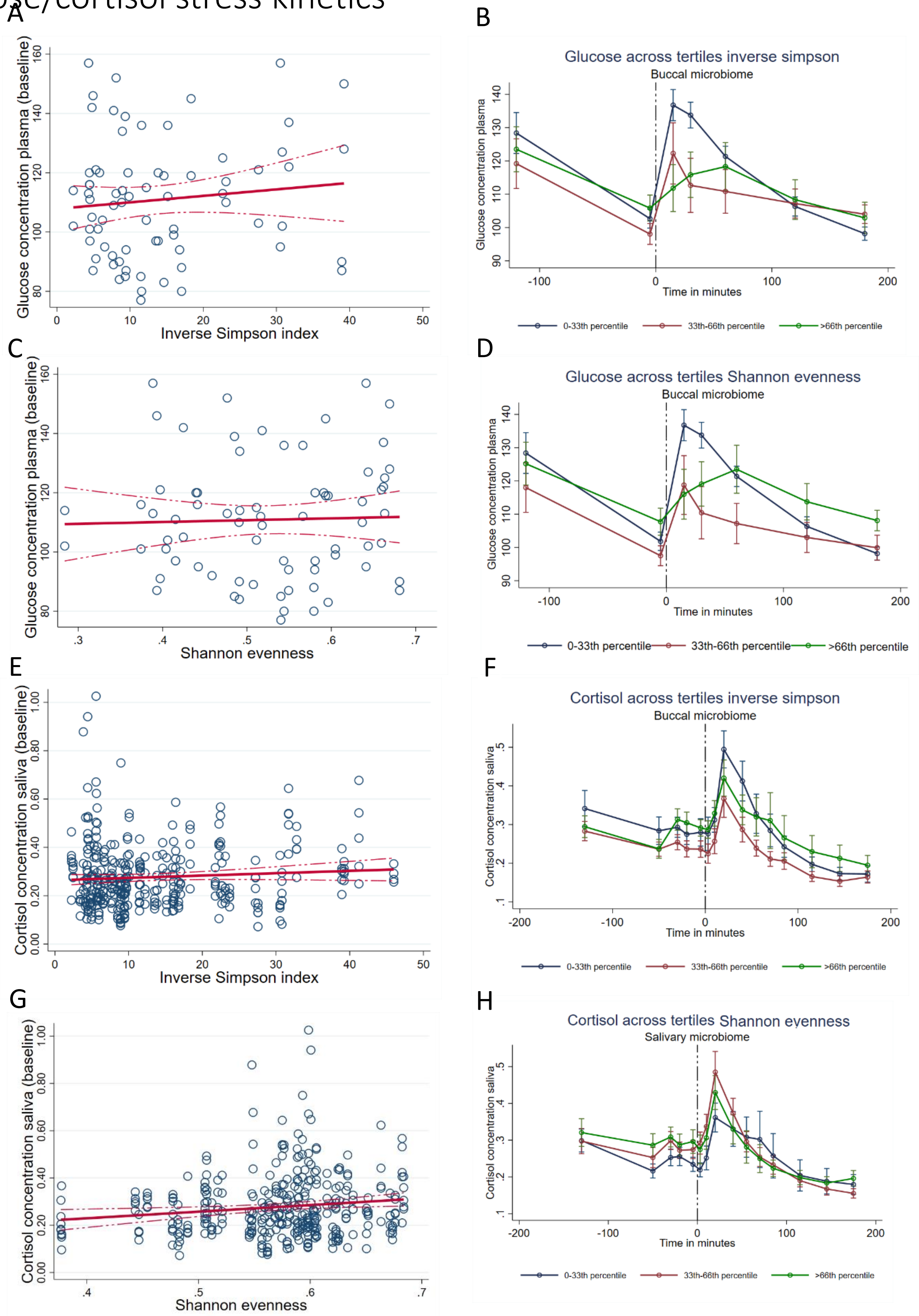

Supp. Fig 2: Divesity indeces are associated with both glucose and cortisol kinetics. Time series of plasma glucose measurements with standard errors as the error bars depending on the diversity of the buccal composition A) *Glucose baseline and diversity score*, B) *Glucose kinetics* across tertiles of inverse simpson diversity index, C) *Glucose baseline and eveness score*, D) *Glucose kinetics* across tertiles of Shannon evenness index. Time series of salivary cortisol measurements with standard errors as the error bars depending on the diversity of the buccal and salivary composition E) *Cortisol baseline and diversity score (buccal microbiome)*, F) Cortisol *kinetics* across tertiles of inverse simpson diversity index, E) *Cortisol baseline and diversity score (buccal microbiome)*, F) Cortisol *kinetics* across tertiles of inverse simpson diversity index *(buccal microbiome)*, G) *Cortisol baseline and evenness score (salivary microbiome)*, H) Cortisol *kinetics* across tertiles of shannon evenness index *(salivary*

Supplementary Table 1A: Summary cortisol and glucose measurements

	Cortisol	Glucose
**mean baseline**	0,265	101,786
**sd baseline**	0,136	12,635
**mean max**	0,545	135,952
**sd max**	0,287	21,664

Supplementary Table 1B: Cohort descriptive table

	Sub cohort		Full cohort		2-way ANOVA p-value	Post-hoc
	Controls	ELA	Controls	ELA		
**Age (years)**	22 (21-23)	23.5 (20-24)	22 (21-23)	24 (20-25)	0.052	Effect of ELA irrespective of cohort as per Elwenspoek 2018
**Sex (female)**	60%	60%	57.5%	68.9%	>0.1	N/A
**Smoking**	15%	30%	14.3%	31.7%	0.07	Effect of ELA irrespective of cohort as per Elwenspoek 2018
**BMI**	22.2 (20.9-24.3	23.7 (22.8-26.2)	22.5 (20.6-24.3)	23.0 (21.1-26.2)	>0.1	N/A
**Age at adoption (Months)**	N/A	5.0 (3-17)	N/A	4.3 (1-19)	>0.1*	*Student's t-test

Elwenspoek MMC (2018). Phenotype and Mechanisms of Altered Immune Functions induced by Early Life Adversity. Department of Psychobiology. Trier, Germany, Univeritv of Trier. **PhD**. https://doi.org/10.25353/ubtr-xxxx-03d5-efe3

Supplementary Table 2A: Summary statistics for associations of genus presences (buccal microbiome) with cortisol at baseline

Genus	b (main effect)	95%- Confidence interval	p-value (main effect)	FDR (main effect)
Caulobacter	-0.05	(-.12519694;.0155708149)	1.25E-01	0.965071519
Solobacterium	0.05	(-.0152609892;.1205356664)	1.26E-01	0.965071519
Tannerella	-0.04	(-.1100842306;.022322394)	1.90E-01	0.965071519
Afipia	0.04	(-.0267690013;.100118186)	2.52E-01	0.965071519
Sphingomonas	0.04	(-.0326581911;.1123641867)	2.76E-01	0.965071519
**Clostridia_UCG-014_ge**	**-0.04**	**(-.1029125874;.0312015353)**	**2.89E-01**	0.965071519
Pseudomonas	0.04	(-.0306278326;.1008468023)	2.90E-01	0.965071519
**Acinetobacter**	**0.04**	**(-.0324617237;.1066313841)**	**2.91E-01**	0.965071519
Catonella	0.03	(-.0307755457;.0999489263)	2.94E-01	0.965071519
Chitinophagaceae_unclassified	0.03	(-.0334707487;.0993231453)	3.26E-01	0.965071519
**Absconditabacteriales_(SR1)_ge**	**-0.03**	**(-.1018826209;.0412417852)**	**4.00E-01**	0.965071519
Methylobacterium-Methylorubrum	0.03	(-.0405320085;.0979963325)	4.10E-01	0.965071519
Paucibacter	0.03	(-.0437309336;.1003278778)	4.35E-01	0.965071519
Microbacteriaceae_unclassified	-0.03	(-.0998519727;.0467986511)	4.72E-01	0.965071519
Bradyrhizobium	-0.02	(-.0888431296;.0427636687)	4.87E-01	0.965071519
Megasphaera	0.02	(-.0486669883;.0886996927)	5.62E-01	0.965071519
Treponema	-0.02	(-.081574382;.0471054458)	5.94E-01	0.965071519
Pseudarcicella	-0.02	(-.0921043264;.0550717886)	6.17E-01	0.965071519
Staphylococcus	-0.02	(-.0886717291;.0535458465)	6.23E-01	0.965071519
Olsenella	0.01	(-.0539806021;.0812130022)	6.89E-01	0.965071519
Anaerovoracaceae_unclassified	0.01	(-.0543361957;.079965807)	7.04E-01	0.965071519
Anaerovoracaceae_ge	-0.01	(-.0755059688;.0545926827)	7.49E-01	0.965071519
uncultured	-0.01	(-.0747556338;.0546871406)	7.58E-01	0.965071519
Fluviicola	-0.01	(-.0843256437;.0637965064)	7.83E-01	0.965071519
Lactobacillus	0.01	(-.0593760368;.0763996744)	8.03E-01	0.965071519
Kingella	-0.01	(-.0795164541;.0636122127)	8.25E-01	0.965071519
Terrisporobacter	-0.01	(-.0688984077;.0583180275)	8.69E-01	0.965071519
Cardiobacterium	0.00	(-.0743750162;.0645947837)	8.89E-01	0.952106352
Oxalobacteraceae_unclassified	0.00	(-.0665516653;.0615041208)	9.37E-01	0.969808842
Fretibacterium	0.00	(-.0749410956;.0775815369)	9.73E-01	0.972518861

Estimates derived from linear regressions adjusted for age, sex, BMI, study group

Supplementary Table 2B: Summary statistics for associations of genus presences (salivary microbiome) with cortisol at baseline

Genus	b (main effect)	95%- Confidence interval	p-value (main effect)	FDR (main effect)
Psychrobacter	-0.07	(-.1303649476;-.0013964502)	4.54E-02	0.812413793
Microbacteriaceae_unclassified	0.06	(-.0102277312;.1217925063)	9.62E-02	0.812413793
Fluviicola	0.05	(-.0107681482;.1150138799)	1.03E-01	0.812413793
JGI_0000069-P22_ge	-0.05	(-.1112505496;.0185416555)	1.58E-01	0.812413793
Chitinophagaceae_unclassified	-0.04	(-.1076462336;.0207020439)	1.81E-01	0.812413793
**Cardiobacterium**	**0.05**	**(-.0234345682;.1205124492)**	**1.83E-01**	0.812413793
Catonella	-0.04	(-.1140735196;.0272501554)	2.24E-01	0.812413793
uncultured	-0.04	(-.1043614843;.0261659362)	2.36E-01	0.812413793
Legionella	-0.04	(-.1083868265;.029135263)	2.54E-01	0.812413793
Xanthobacteraceae_unclassified	0.04	(-.0273560301;.0996062477)	2.60E-01	0.812413793
Absconditabacteriales_(SR1)_ge	-0.03	(-.0999197987;.0332557758)	3.21E-01	0.812413793
Treponema	0.03	(-.0327319237;.0949672676)	3.34E-01	0.812413793
Lactobacillus	0.03	(-.039388421;.107522599)	3.58E-01	0.812413793
Mycobacterium	-0.03	(-.1052742033;.0399786783)	3.73E-01	0.812413793
Alysiella	0.03	(-.0361157656;.0928629601)	3.83E-01	0.812413793
Atopobium	0.03	(-.0377797545;.094537102)	3.95E-01	0.812413793
Lachnoanaerobaculum	0.03	(-.0409801637;.0934404939)	4.38E-01	0.812413793
Enterococcaceae_unclassified	-0.03	(-.0946371982;.0421164189)	4.46E-01	0.812413793
Clostridia_UCG-014_ge	-0.02	(-.0874716477;.0420199368)	4.86E-01	0.812413793
Rhodocyclaceae_unclassified	-0.02	(-.0907746375;.043996887)	4.91E-01	0.812413793
Solobacterium	-0.02	(-.0839258123;.0430709787)	5.23E-01	0.812413793
Veillonellaceae_unclassified	-0.02	(-.0842644086;.0449497487)	5.45E-01	0.812413793
Bifidobacterium	-0.02	(-.0959776283;.0520427621)	5.55E-01	0.812413793
Butyrivibrio	0.02	(-.0518418024;.0950412305)	5.59E-01	0.812413793
Prevotellaceae_ge	-0.02	(-.0853214845;.0476356146)	5.73E-01	0.812413793
Parcubacteria_ge	0.02	(-.0496299637;.0869336981)	5.87E-01	0.812413793
Caulobacter	0.02	(-.0496687343;.0857674754)	5.96E-01	0.812413793
Candidatus_Fritschea	0.02	(-.0504067377;.0856714913)	6.06E-01	0.812413793
Kingella	-0.02	(-.0890375974;.053399745)	6.19E-01	0.812413793
Tannerella	-0.01	(-.0825870572;.05285572)	6.62E-01	0.836
Haemophilus	-0.01	(-.0778734457;.0529201282)	7.04E-01	0.858064516
**Campylobacter**	**-0.01**	**(-.0829842355;.0669805522)**	**8.32E-01**	0.95542857
Staphylococcus	0.01	(-.057389864;.0705888389)	8.37E-01	0.95542857
Anaerovoracaceae_ge	0.01	(-.0612909419;.0734088198)	8.58E-01	0.95542857
Parvimonas	0.00	(-.0703961013;.0605362171)	8.81E-01	0.95542857
Candidatus_Peribacteria_ge	0.00	(-.060379801;.0656462656)	9.34E-01	0.9816666
Terrisporobacter	0.00	(-.0639913451;.0651422587)	9.86E-01	1
Anaerovoracaceae_unclassified	0.00	(-.0648173144;.0647551765)	9.99E-01	1

Estimates derived from linear regressions adjusted for age, sex, BMI, study group

Supplementary Table 3A: Summary statistics for associations of genus presences (buccal microbiome) with cortisol kinetics

Genus	b (main effect)	95%- Confidence interval	p-value (main effect)	FDR (main effect)	p-value (interaction effect)	FDR (interaction effect)
**Absconditabacteriales_(SR1)_ge**	**0,066312537**	**(.00966736;.1229577066)**	**0,024201984**	**0,726059508**	**1,88775E-07**	**5,66324E-06**
**Acinetobacter**	**-0,002167796**	**(-.0593392206;.0550036297)**	**0,940759122**	**0,979221642**	**0,0026098**	**0,033412639**
**Clostridia_UCG-014_ge**	**0,006941371**	**(-.0481257662;.0620085082)**	**0,804901659**	**0,979221642**	**0,003341264**	**0,033412639**
Tannerella	-0,018204354	(-.0722595071;.035850798)	0,509875834	0,951675203	0,086348303	0,64761227
Anaerovoracaceae_ge	-0,030020241	(-.0824958019;.0224553195)	0,264315188	0,935363372	0,10917411	0,65504466
Fluviicola	0,020780789	(-.0385686758;.0801302536)	0,49322471	0,951675203	0,305241287	0,999979854
Treponema	-0,010057595	(-.0622454512;.0421302612)	0,705781162	0,951675203	0,318851382	0,999979854
Fretibacterium	-0,037569832	(-.098991546;.0238518802)	0,232934698	0,935363372	0,331063688	0,999979854
Pseudarcicella	-0,022432623	(-.0810773549;.0362121081)	0,454332083	0,951675203	0,423401058	0,999979854
Pseudomonas	0,023725929	(-.029690512;.0771423683)	0,385266632	0,951675203	0,472362995	0,999979854
Oxalobacteraceae_unclassified	-0,004051932	(-.0556805549;.0475766913)	0,877760231	0,979221642	0,472631514	0,999979854
Solobacterium	0,031407386	(-.0238763522;.0866911255)	0,26757732	0,935363372	0,504407823	0,999979854
Olsenella	-0,021927347	(-.0768540551;.0329993598)	0,434946448	0,951675203	0,506147802	0,999979854
Bradyrhizobium	-0,033726849	(-.0869117541;.0194580593)	0,216361865	0,935363372	0,665236771	0,999979854
uncultured	0,010173948	(-.0424366004;.0627844977)	0,704810262	0,951675203	0,680109143	0,999979854
Caulobacter	-0,052983027	(-.110044259;.0040782024)	0,071979478	0,935363372	0,786419809	0,999979854
Terrisporobacter	-0,00068601	(-.0523105237;.0509385032)	0,979221642	0,979221642	0,851535678	0,999979854
Sphingomonas	0,015996905	(-.0434937437;.0754875539)	0,598519385	0,951675203	0,876023114	0,999979854
Kingella	0,006142237	(-.0522810955;.0645655699)	0,836770654	0,979221642	0,935832679	0,999979854
Megasphaera	0,040590297	(-.0145587104;.0957393073)	0,152075037	0,935363372	0,955122769	0,999979854
Afipia	0,011561705	(-.0403211033;.0634445131)	0,662502706	0,951675203	0,980777621	0,999979854
Anaerovoracaceae_unclassified	0,030712068	(-.0235784177;.085002553)	0,269577205	0,935363372	0,993454039	0,999979854
Catonella	0,018580532	(-.0343995637;.0715606277)	0,49255991	0,951675203	0,993897438	0,999979854
Chitinophagaceae_unclassified	0,009607429	(-.0448486769;.064063535)	0,729617655	0,951675203	0,994079411	0,999979854
Paucibacter	0,016223092	(-.042739384;.0751855669)	0,590077817	0,951675203	0,994134784	0,999979854
Methylobacterium-Methylorubrum	0,010824515	(-.0458822657;.0675312968)	0,708442152	0,951675203	0,996311128	0,999979854
Lactobacillus	-0,002406226	(-.0578055783;.0529931251)	0,93216002	0,979221642	0,998978674	0,999979854
Microbacteriaceae_unclassified	-0,03243845	(-.0911223953;.0262454958)	0,280609012	0,935363372	0,999470651	0,999979854
Staphylococcus	-0,022683131	(-.0803616162;.0349953549)	0,441773295	0,951675203	0,999968886	0,999979854
Cardiobacterium	-0,001315038	(-.0578480511;.0552179749)	0,96363616	0,979221642	0,999979854	0,999979854

Estimates derived from linear regressions adjusted for age, sex, BMI, study group

*p value is derived by (LR) Likelihood Ratio test

Supplementary Table3B: Summary statistics for associations of genus presences (salivary microbiome) with cortisol kinetics

Genus	b (main effect)	95%- Confidence interval	p-value (main effect)	FDR (main effect)	p-value (interaction effect)	FDR (interaction effect)
**Campylobacter**	**-0,063257799**	**(-.1218681816;-.0046474102)**	**0,037219156**	**0,86270454**	**3,4318E-06**	**0,000130408**
**Cardiobacterium**	**-0,007194403**	**(-.0664722093;.0520834035)**	**0,81201148**	**0,975245991**	**0,000396326**	**0,00753019**
Rhodocyclaceae_unclassified	-0,036371656	(-.0900312449;.01728793)	0,186733887	0,889652872	0,006187909	0,078380178
Lactobacillus	0,016379435	(-.0421834164;.0749422846)	0,583958507	0,975245991	0,013397842	0,127279496
Microbacteriaceae_unclassified	0,05056522	(-.0031145544;.1042449916)	0,068108253	0,86270454	0,074640624	0,564438939
Kingella	0,011769122	(-.0455649927;.0691032357)	0,687610626	0,975245991	0,089121938	0,564438939
Alysiella	-0,031289119	(-.0833454293;.0207671927)	0,241023928	0,889652872	0,170741752	0,838757109
Legionella	-0,053374495	(-.1077620864;.0010130982)	0,057568334	0,86270454	0,176580444	0,838757109
Catonella	-0,02953266	(-.0872027388;.0281374194)	0,317221284	0,889652872	0,248465374	0,972025311
Caulobacter	-0,012495148	(-.0675732528;.0425829577)	0,656798363	0,975245991	0,255796134	0,972025311
Psychrobacter	-0,039454747	(-.092373228;.0134637321)	0,146925449	0,889652872	0,422363281	0,999092817
Chitinophagaceae_unclassified	-0,022675389	(-.0753853275;.0300345487)	0,400342524	0,889652872	0,491223067	0,999092817
Staphylococcus	0,019612441	(-.0321044368;.0713293183)	0,458169937	0,889652872	0,510737538	0,999092817
Solobacterium	0,004039554	(-.0477404389;.0558195472)	0,878482997	0,975245991	0,533118665	0,999092817
Bifidobacterium	0,005738492	(-.0547958407;.0662728247)	0,852617919	0,975245991	0,564693213	0,999092817
Absconditabacteriales_(SR1)_ge	0,034798697	(-.0192227651;.0888201603)	0,209247902	0,889652872	0,568065405	0,999092817
Parvimonas	0,008768397	(-.0444788711;.0620156662)	0,746973395	0,975245991	0,618783772	0,999092817
uncultured	-0,020193122	(-.0738164708;.033430225)	0,461320698	0,889652872	0,733541131	0,999092817
Prevotellaceae_ge	-0,011177747	(-.0652507262;.0428952323)	0,685527444	0,975245991	0,789423585	0,999092817
Mycobacterium	0,000802164	(-.0577543466;.0593586751)	0,978579819	0,978579819	0,800130486	0,999092817
Haemophilus	-0,00500164	(-.0578127485;.0478094676)	0,852756619	0,975245991	0,86424619	0,999092817
Xanthobacteraceae_unclassified	0,021401266	(-.030504592;.0733071244)	0,420077741	0,889652872	0,86993593	0,999092817
Butyrivibrio	-0,008645127	(-.0687190586;.0514288056)	0,777961016	0,975245991	0,89323765	0,999092817
Parcubacteria_ge	-0,001800277	(-.0576015291;.0540009751)	0,949581623	0,975245991	0,907996178	0,999092817
Lachnoanaerobaculum	0,020272134	(-.0344052309;.0749494985)	0,468238354	0,889652872	0,910029948	0,999092817
Candidatus_Fritschea	-0,003531496	(-.0589490107;.0518860189)	0,900608063	0,975245991	0,928387523	0,999092817
JGI_0000069-P22_ge	-0,026497822	(-.0798503091;.0268546645)	0,33194527	0,889652872	0,946327329	0,999092817
Clostridia_UCG-014_ge	-0,008557531	(-.0612974514;.0441823902)	0,750551045	0,975245991	0,955659032	0,999092817
Anaerovoracaceae_ge	-0,004563729	(-.0594582206;.0503307621)	0,870573938	0,975245991	0,957597375	0,999092817
Candidatus_Peribacteria_ge	0,002182805	(-.0488542283;.053219838)	0,933196664	0,975245991	0,966054559	0,999092817
Tannerella	-0,02765378	(-.0824958546;.0271882958)	0,324639797	0,889652872	0,975851238	0,999092817
Fluviicola	0,028372029	(-.0230955522;.0798396113)	0,281898439	0,889652872	0,977157116	0,999092817
Enterococcaceae_unclassified	-0,036617067	(-.0917439716;.0185098347)	0,195584014	0,889652872	0,979837477	0,999092817
Treponema	0,022297088	(-.0297945844;.0743887613)	0,402693301	0,889652872	0,980116129	0,999092817
Anaerovoracaceae_unclassified	-0,00250341	(-.0552644156;.0502575958)	0,925908387	0,975245991	0,982242525	0,999092817
Veillonellaceae_unclassified	-0,025425831	(-.0778970722;.0270454121)	0,343761593	0,889652872	0,984011829	0,999092817
Atopobium	0,032671351	(-.0207134793;.0860561806)	0,232657254	0,889652872	0,994564295	0,999092817
Terrisporobacter	0,013965657	(-.0385198027;.0664511168)	0,602343559	0,975245991	0,999092817	0,999092817

Estimates derived from linear regressions adjusted for age, sex, BMI, study group

*p value is derived by (LR) Likelihood Ratio test

Supplementary Table 4A: Summary statistics for associations of genus presences (buccal microbiome) with glucose at baseline

Genus	b (main effect)	95%- Confidence interval	p-value (main effect)	FDR (main effect)
Cardiobacterium	9,00	(1.112998687;16.88911102)	2,65E-02	0,794168953
Microbacteriaceae_unclassified	-8,92	(-17.23567294;-.6014037917)	3,63E-02	0,544494297
**Absconditabacteriales_(SR1)_ge**	**7,61**	**(-.7310975565;15.9601094)**	**7,25E-02**	**0,724722445**
uncultured	5,67	(-1.882635383;13.21733499)	1,37E-01	1,024885252
Pseudarcicella	-6,02	(-14.84764513;2.812167422)	1,75E-01	1,052592337
Pseudomonas	4,98	(-2.564502095;12.51713235)	1,89E-01	0,94583258
Terrisporobacter	4,80	(-2.51174851;12.10952311)	1,91E-01	0,820552749
Afipia	-4,33	(-11.81853664;3.168017104)	2,49E-01	0,935358871
Chitinophagaceae_unclassified	-4,24	(-11.86553311;3.391480538)	2,67E-01	0,891435345
Anaerovoracaceae_ge	3,94	(-3.778008529;11.65295193)	3,08E-01	0,922707886
Staphylococcus	-4,04	(-12.36251518;4.278236761)	3,31E-01	0,902907713
Catonella	3,31	(-4.210954832;10.82955437)	3,78E-01	0,945175663
Lactobacillus	-3,60	(-12.11691318;4.913195505)	3,97E-01	0,91530809
**Oxalobacteraceae_unclassified**	**3,16**	**(-4.551490279;10.87265744)**	**4,11E-01**	**0,881491516**
Megasphaera	3,11	(-4.950087836;11.16251869)	4,39E-01	0,878703654
Fretibacterium	-2,73	(-11.89952922;6.432131508)	5,49E-01	1,029477865
Clostridia_UCG-014_ge	-2,26	(-9.973906375;5.44930463)	5,56E-01	0,980446584
Anaerovoracaceae_unclassified	2,34	(-5.732742995;10.41146288)	5,60E-01	0,933939815
Methylobacterium-Methylorubrum	2,29	(-5.644874302;10.23251679)	5,62E-01	0,886627906
Olsenella	-1,97	(-10.57924998;6.640666582)	6,46E-01	0,968299538
Bradyrhizobium	-1,56	(-8.951387658;5.832265189)	6,71E-01	0,958964995
Kingella	-1,75	(-10.1489825;6.643199741)	6,75E-01	0,919788724
Solobacterium	1,63	(-6.455771414;9.710325486)	6,85E-01	0,894098204
Paucibacter	-1,27	(-9.194255015;6.65262782)	7,47E-01	0,933566093
Caulobacter	-1,32	(-9.840376828;7.195751174)	7,55E-01	0,905646515
**Sphingomonas**	**0,97**	**(-7.13232319;9.065517435)**	**8,10E-01**	**0,934747435**
Fluviicola	-0,95	(-9.908223573;8.012730983)	8,31E-01	0,923732519
Tannerella	0,81	(-6.861434162;8.474326394)	8,32E-01	0,891747198
Treponema	-0,67	(-8.38395426;7.051263115)	8,62E-01	0,891699647
Acinetobacter	0,43	(-7.437201081;8.301330124)	9,12E-01	0,91195482

Estimates derived from linear regressions adjusted for age, sex, BMI, study group

Supplementary Table 4B: Summary statistics for associations of genus presences (salivary microbiome) with cortisol at baseline

Genus	b (main effect)	95%- Confidence interval	p-value (main effect)	FDR (main effect)
Terrisporobacter	10,50	(3.849105618;17.14352736)	2,85E-03	0,108232221
Atopobium	11,09	(3.540405372;18.63633989)	5,15E-03	0,097796111
Anaerovoracaceae_ge	9,97	(2.684302454;17.25402885)	8,69E-03	0,110071028
Solobacterium	7,95	(.9345075675;14.96841377)	2,75E-02	0,260936246
Tannerella	7,62	(.286354168;14.95141418)	4,21E-02	0,320135516
Butyrivibrio	7,14	(-.6252242706;14.9051317)	7,04E-02	0,445727557
Legionella	-6,83	(-14.51376264;.8500888909)	7,97E-02	0,432429233
Lachnoanaerobaculum	6,95	(-1.116670243;15.02519287)	8,91E-02	0,423121145
Anaerovoracaceae_unclassified	6,80	(-1.135552045;14.73731101)	9,08E-02	0,383271365
Absconditabacteriales_(SR1)_ge	6,72	(-1.173108964;14.60656607)	9,28E-02	0,35272748
Psychrobacter	-5,97	(-14.03195871;2.08239564)	1,41E-01	0,488206779
Microbacteriaceae_unclassified	-6,11	(-14.52549516;2.300804675)	1,49E-01	0,472842912
JGI_0000069-P22_ge	5,34	(-2.40966649;13.09771739)	1,71E-01	0,499041097
Rhodocyclaceae_unclassified	4,84	(-3.215327243;12.88536552)	2,31E-01	0,627328883
Bifidobacterium	5,11	(-3.732401693;13.95186873)	2,49E-01	0,630529934
Parcubacteria_ge	-3,94	(-11.80179175;3.917309936)	3,16E-01	0,750063602
Staphylococcus	3,54	(-3.693253532;10.76740458)	3,28E-01	0,732617175
Kingella	-3,58	(-11.50817235;4.344357598)	3,66E-01	0,771615962
Haemophilus	3,15	(-4.495597753;10.7947807)	4,09E-01	0,817878306
Candidatus_Peribacteria_ge	-2,92	(-10.36616317;4.527128334)	4,32E-01	0,820322904
Enterococcaceae_unclassified	-3,46	(-12.30118005;5.382736088)	4,33E-01	0,783010088
Cardiobacterium	3,21	(-5.492152086;11.91207437)	4,59E-01	0,793260043
Caulobacter	2,30	(-5.569723578;10.17464755)	5,57E-01	0,919881505
uncultured	-1,95	(-10.02152598;6.113069847)	6,26E-01	0,991491954
Alysiella	1,68	(-6.072932737;9.439621128)	6,62E-01	1,006931362
Treponema	1,56	(-6.159589998;9.270458443)	6,85E-01	1,001220405
Clostridia_UCG-014_ge	-1,44	(-9.090554067;6.220144861)	7,06E-01	0,993650812
Chitinophagaceae_unclassified	1,37	(-5.970159744;8.707191704)	7,08E-01	0,960183804
Veillonellaceae_unclassified	-1,44	(-9.151671905;6.277041308)	7,08E-01	0,927393066
Fluviicola	1,32	(-5.999572875;8.629983763)	7,18E-01	0,908837676
Candidatus_Fritschea	1,30	(-6.858172083;9.451450422)	7,49E-01	0,91807973
Lactobacillus	1,23	(-7.186546581;9.642789658)	7,69E-01	0,913104963
Mycobacterium	0,90	(-7.751066358;9.560272386)	8,33E-01	0,959600123
Parvimonas	0,47	(-7.411109889;8.346559847)	9,05E-01	1,011289425
Catonella	0,51	(-8.779428434;9.798505686)	9,12E-01	0,990211099
Campylobacter	0,43	(-8.234571845;9.099292614)	9,20E-01	0,971083446
Prevotellaceae_ge	-0,25	(-8.029761209;7.537820264)	9,49E-01	0,974909857
Xanthobacteraceae_unclassified	-0,12	(-8.180597841;7.946490611)	9,77E-01	0,976675808

Estimates derived from linear regressions adjusted for age, sex, BMI, study group

Supplementary Table 5A: Summary statistics for associations of genus presences (buccal microbiome) with glucose at baseline

Genus	b (main effect)	95%- Confidence interval	p-value (main effect)	FDR (main effect)	p-value (interaction effect)	FDR (interaction effect)
**Absconditabacteriales_(SR1)_ge**	**1,108118892**	**(-8.040347552;10.25658538)**	**0,812347293**	**0,879204561**	**0,000162109**	**0,004863267**
**Oxalobacteraceae_unclassified**	**-1,106256485**	**(-9.24642054;7.033907614)**	**0,790110409**	**0,879204561**	**0,0031046**	**0,034873711**
**Sphingomonas**	**-1,582074523**	**(-10.11226861;6.948119481)**	**0,716627061**	**0,879204561**	**0,003487371**	**0,034873711**
Anaerovoracaceae_ge	-1,90981555	(-10.07152474;6.25189357)	0,647015154	0,879204561	0,032191046	0,241432842
Lactobacillus	-7,140242577	(-15.84291761;1.562432068)	0,113857418	0,64581804	0,063482419	0,325043691
Afipia	1,875171065	(-6.175176904;9.925518953)	0,648256481	0,879204561	0,080665492	0,325043691
Acinetobacter	2,767563105	(-5.36007483;10.89520124)	0,505572855	0,879204561	0,094126277	0,325043691
Treponema	0,042189714	(-8.022246526;8.106625953)	0,991818726	0,991818726	0,101445042	0,325043691
Paucibacter	-1,848830581	(-10.27632301;6.578661923)	0,66779089	0,879204561	0,119578123	0,325043691
Methylobacterium-Methylorubrum	2,025284529	(-6.381589741;10.43215877)	0,637256145	0,879204561	0,129042566	0,325043691
Bradyrhizobium	-0,994610012	(-8.729807829;6.740587786)	0,801180005	0,879204561	0,136250123	0,325043691
Microbacteriaceae_unclassified	-10,80539703	(-19.33025747;-2.280537469)	0,016681703	0,500451084	0,150595576	0,325043691
Staphylococcus	-0,847890258	(-9.626540898;7.930760392)	0,849897742	0,879204561	0,150931537	0,325043691
Anaerovoracaceae_unclassified	-4,36980629	(-12.68871149;3.949099142)	0,306937248	0,879204561	0,151687056	0,325043691
Tannerella	-3,683117867	(-11.68163339;4.315397458)	0,370070219	0,879204561	0,192679226	0,364549421
Caulobacter	3,607959986	(-5.189520588;12.40544048)	0,423387885	0,879204561	0,194426358	0,364549421
Chitinophagaceae_unclassified	-2,097111702	(-10.13930373;5.945080106)	0,609909177	0,879204561	0,30250743	0,533836642
Fluviicola	-3,63654685	(-12.88607196;5.612978203)	0,442570597	0,879204561	0,324906975	0,541511625
Catonella	-1,614660263	(-9.486380029;6.257059467)	0,688092172	0,879204561	0,383927345	0,595555561
Terrisporobacter	-3,149969816	(-10.92514449;4.625204785)	0,429357827	0,879204561	0,405572236	0,595555561
Fretibacterium	3,508980513	(-6.039287655;13.05724885)	0,472631067	0,879204561	0,416888893	0,595555561
Solobacterium	-3,199092627	(-11.66578207;5.267596866)	0,460298747	0,879204561	0,444467127	0,606091537
Pseudarcicella	-8,59676075	(-17.88034826;.686825907)	0,0757671	0,64581804	0,526688814	0,68698541
Olsenella	-6,868649006	(-15.60720053;1.869902713)	0,129163608	0,64581804	0,594035745	0,694963733
Pseudomonas	7,073961735	(-.7424278197;14.89035116)	0,081686825	0,64581804	0,59484458	0,694963733
Cardiobacterium	0,853375196	(-7.935659364;9.642409789)	0,849106908	0,879204561	0,603420734	0,694963733
uncultured	-2,844639063	(-10.91942945;5.230151461)	0,491473585	0,879204561	0,62546736	0,694963733
Clostridia_UCG-014_ge	-6,206682682	(-14.07015648;1.656791555)	0,12672548	0,64581804	0,678139627	0,726578172
Megasphaera	1,262823224	(-7.170566775;9.696213312)	0,769213438	0,879204561	0,763706267	0,790040966
Kingella	2,327854156	(-6.421659025;11.0773675)	0,602672279	0,879204561	0,91031146	0,91031146

Estimates derived from linear regressions adjusted for age, s

*p value is derived by (LR) Likelihood Ratio test

Supplementary Table 5B: Summary statistics for associations of genus presences salivary microbiome) with glucose at baseline

Genus	b (main effect)	95%- Confidence interval	p-value (main effect)	FDR (main effect)	p-value (interaction effect)	FDR (interaction effect)
Veillonellaceae_unclassified	-8,968733788	(-16.5142991;-1.423169199)	0,024279365	0,461307941	0,014283045	0,485814916
Xanthobacteraceae_unclassified	6,724564552	(-1.588636457;15.03776521)	0,118424296	0,900024652	0,0338687	0,485814916
Clostridia_UCG-014_ge	2,806957245	(-5.146401512;10.76031581)	0,490610808	0,998769104	0,049233787	0,485814916
Alysiella	-2,144349813	(-10.21994384;5.931244416)	0,603479445	0,998769104	0,051138412	0,485814916
Prevotellaceae_ge	-1,38239181	(-9.636497753;6.871714075)	0,742840111	0,998769104	0,127450183	0,809536657
Caulobacter	-3,676433802	(-11.97419499;4.621327316)	0,387727499	0,998769104	0,141235828	0,809536657
Candidatus_Peribacteria_ge	-9,501239777	(-16.759675;-2.242805502)	0,013562188	0,461307941	0,149125174	0,809536657
Bifidobacterium	2,873684406	(-6.470332472;12.2177011)	0,547958136	0,998769104	0,229177058	0,937643132
Solobacterium	-2,37242341	(-10.3094694;5.564622643)	0,558659732	0,998769104	0,261545449	0,937643132
Catonella	5,542924881	(-3.903017899;14.98886729)	0,253890961	0,998769104	0,268142819	0,937643132
Microbacteriaceae_unclassified	-0,941047668	(-9.93229209;8.050196717)	0,837521374	0,998769104	0,271423012	0,937643132
Staphylococcus	-4,218185902	(-11.76792758;3.331556228)	0,276827037	0,998769104	0,342034519	0,937690969
uncultured	-2,281271935	(-10.73727606;6.174732425)	0,597496212	0,998769104	0,343019038	0,937690969
Atopobium	-1,469155669	(-10.51600543;7.577694148)	0,750339568	0,998769104	0,345465094	0,937690969
Tannerella	1,698717356	(-6.521723232;9.919158047)	0,685851872	0,998769104	0,426901817	0,995857426
Parcubacteria_ge	-0,201737985	(-8.527780351;8.124304386)	0,962124884	0,998769104	0,43016246	0,995857426
JGI_0000069-P22_ge	0,006528143	(-8.287157469;8.300213756)	0,998769104	0,998769104	0,446743816	0,995857426
Lachnoanaerobaculum	0,745593429	(-8.225388942;9.716575782)	0,870648026	0,998769104	0,476378113	0,995857426
Kingella	-3,284747124	(-11.63978279;5.070288517)	0,442360163	0,998769104	0,500414908	0,995857426
Anaerovoracaceae_ge	7,59585762	(-.747432048;15.93914714)	0,081013314	0,769626487	0,548102677	0,995857426
Chitinophagaceae_unclassified	0,698210359	(-7.01015923;8.406579973)	0,859116375	0,998769104	0,550342262	0,995857426
Absconditabacteriales_(SR1)_ge	7,875743389	(-.2972278104;16.04871491)	0,06430506	0,769626487	0,607836783	0,998137176
Cardiobacterium	-2,627762794	(-11.69783581;6.442310434)	0,570941627	0,998769104	0,682448328	0,998137176
Treponema	1,882017374	(-6.195317568;9.959352302)	0,64810884	0,998769104	0,684562027	0,998137176
Terrisporobacter	2,380518675	(-5.512972912;10.27401049)	0,555486917	0,998769104	0,684651971	0,998137176
Candidatus_Fritschea	1,323800445	(-7.426631812;10.07423275)	0,766902566	0,998769104	0,711738229	0,998137176
Mycobacterium	4,11980772	(-4.862002795;13.10161823)	0,370829225	0,998769104	0,733943164	0,998137176
Campylobacter	0,03092074	(-8.98326165;9.045103131)	0,994635761	0,998769104	0,801504314	0,998137176
Anaerovoracaceae_unclassified	-0,26436764	(-8.836094674;8.30735937)	0,951800346	0,998769104	0,884413064	0,998137176
Butyrivibrio	3,856467247	(-4.77545284;12.48838735)	0,383675516	0,998769104	0,89325273	0,998137176
Haemophilus	4,067944527	(-3.959986094;12.09587553)	0,323215127	0,998769104	0,893303573	0,998137176
Parvimonas	-1,451659203	(-9.622101986;6.718783665)	0,727915466	0,998769104	0,943605244	0,998137176
Rhodocyclaceae_unclassified	3,423507929	(-5.071900548;11.9189163)	0,431901246	0,998769104	0,965039194	0,998137176
Legionella	-0,593618929	(-8.967850358;7.780612448)	0,889518797	0,998769104	0,965999067	0,998137176
Enterococcaceae_unclassified	-1,160422802	(-10.38610937;8.065263783)	0,805366576	0,998769104	0,981237292	0,998137176
Fluviicola	-0,267041415	(-7.999012482;7.464929662)	0,946035385	0,998769104	0,988658786	0,998137176
Lactobacillus	2,449516773	(-6.31546954;11.21450325)	0,584405482	0,998769104	0,98938942	0,998137176
Psychrobacter	-2,397624969	(-10.98814166;6.192891846)	0,585021913	0,998769104	0,998137176	0,998137176

Estimates derived from linear regressions adjusted for age, sex, BMI, study group

*p value is derived by (LR) Likelihood Ratio test

Supplementary Table 6A: Summary statistics for associations of genus abundances (buccal microbiome) with glucose time series and interaction effects with time

Genus	b (main effect)	95%- Confidence interval	p-value (main effect)	FDR (main effect)	p-value (interaction effect)	FDR (interaction effect)
**Comamonadaceae_unclassified**	**-1363,446533**	**(-3610.827329;883.9342971)**	**0,238618851**	**0,799876892**	**0,000141843**	**0,005828104**
**Sphingomonas**	**-1206,255005**	**(-3533.059996;1120.55005)**	**0,313100874**	**0,799876892**	**0,000253396**	**0,005828104**
**Bradyrhizobium**	**-1774,014771**	**(-5951.095083;2403.065579)**	**0,407501519**	**0,799876892**	**0,00092461**	**0,014177356**
**Methylobacterium-Methylorubrum**	**-1288,639526**	**(-4633.25803;2055.978907)**	**0,45210433**	**0,799876892**	**0,003899289**	**0,03799473**
**Paucibacter**	**-1232,66333**	**(-3473.824753;1008.498114)**	**0,284910351**	**0,799876892**	**0,004129862**	**0,03799473**
**Flavobacterium**	**-1425,428101**	**(-3214.706052;363.8497985)**	**0,124059528**	**0,799876892**	**0,00599251**	**0,045942579**
Neisseria	16,38541794	(-59.82716027;92.59799722)	0,673937023	0,92553178	0,01132971	0,065910432
Prevotella	72,95637512	(-383.0589321;528.9716834)	0,754052758	0,963511858	0,011462684	0,065910432
Acinetobacter	122,6909866	(-1126.189331;1371.571289)	0,847332776	0,979194403	0,018774085	0,078385604
Oxalobacteraceae_unclassified	-1106,142212	(-3778.586685;1566.30228)	0,419124633	0,799876892	0,019104423	0,078385604
Catonella	-417,1212463	(-7884.578753;7050.336285)	0,912834883	0,979194403	0,019546641	0,078385604
Rothia	-64,54467773	(-133.5821427;4.492794473)	0,07271412	0,799876892	0,020448418	0,078385604
Lachnospiraceae_unclassified	181,8621674	(-693.2486007;1056.972937)	0,684088707	0,92553178	0,023990527	0,084889556
Alysiella	417,6565857	(-517.8335863;1353.146755)	0,383602053	0,799876892	0,036529556	0,120025684
Terrisporobacter	1613,942627	(-785.5992086;4013.484577)	0,192218751	0,799876892	0,049705572	0,148127843
Fusobacterium	195,7896729	(-29.38489654;420.9642499)	0,094053179	0,799876892	0,051522728	0,148127843
Veillonella	1,13592732	(-47.05052486;49.32237951)	0,963150978	0,979194403	0,070216447	0,189997445
Pasteurellaceae_unclassified	-331,22995	(-977.7858426;315.3259399)	0,31780538	0,799876892	0,080315575	0,202473072
Corynebacterium	76,42250061	(-632.1544489;784.9994522)	0,832598627	0,979194403	0,083630182	0,202473072
Prevotellaceae_unclassified	418,3184509	(-259.0934283;1095.730355)	0,22984457	0,799876892	0,106482714	0,244910243
Tannerella	1364,830933	(-1411.665776;4141.327716)	0,337877929	0,799876892	0,125408217	0,274703714
Veillonellaceae_unclassified	-395,9350891	(-1293.590358;501.7201884)	0,389519185	0,799876892	0,177978128	0,368079151
Porphyromonas	120,3147278	(-219.8970262;460.5264834)	0,489422172	0,804050712	0,192226842	0,368079151
Treponema	434,7358093	(-1606.635189;2476.106836)	0,676634908	0,92553178	0,199609026	0,368079151
Streptococcus	-5,320678711	(-22.3847821;11.74342492)	0,542231143	0,860090778	0,203648224	0,368079151
Micrococcaceae_unclassified	4,228279591	(-39.12355488;47.58011416)	0,848439991	0,979194403	0,208044738	0,368079151
Streptobacillus	68,22906494	(-74.9231383;211.3812752)	0,353056252	0,799876892	0,250390887	0,426591882
Lachnoanaerobaculum	7,943053722	(-537.1751312;553.0612382)	0,977216423	0,979194403	0,278778642	0,457993484
Planococcaceae_unclassified	1452,634399	(-1186.628809;4091.897723)	0,283701688	0,799876892	0,302815825	0,48032855
Oribacterium	308,2524719	(-275.632784;892.1377462)	0,304106802	0,799876892	0,321971565	0,487743178
Capnocytophaga	761,1723633	(-167.527392;1689.872147)	0,113511205	0,799876892	0,328696489	0,487743178
Lautropia	-21,62424469	(-288.9087283;245.6602396)	0,874014139	0,979194403	0,38122344	0,545805658
Campylobacter	303,0724182	(-975.2810323;1581.425839)	0,642563105	0,92553178	0,391556233	0,545805658
Neisseriaceae_unclassified	211,6434479	(-329.8354007;753.1223089)	0,445126146	0,799876892	0,425417542	0,57556491
Atopobium	-3108,297607	(-6500.210615;283.6153685)	0,078266166	0,799876892	0,442126334	0,581080324
Actinobacillus	-1,453135967	(-33.66338321;30.75711139)	0,929541528	0,979194403	0,473673284	0,605249196
Weeksellaceae_unclassified	1862,910767	(40.78332585;3685.038152)	0,050418261	0,799876892	0,503760934	0,626297377
Selenomonas	89,13140106	(-883.0174149;1061.280215)	0,857399046	0,979194403	0,576115191	0,6974026
Prevotellaceae_ge	2579,496338	(-609.5893741;5768.582031)	0,118648604	0,799876892	0,609468639	0,718860446
Actinomyces	16,05877876	(-23.59624429;55.71380225)	0,428982109	0,799876892	0,635666966	0,731017011
Cardiobacterium	952,2024536	(-414.1424671;2318.547389)	0,17652905	0,799876892	0,660320222	0,740847079
Parvimonas	141,6492615	(-205.0171351;488.315664)	0,424528927	0,799876892	0,805963755	0,882722207
Alloprevotella	153,0124664	(-550.0960414;856.1209613)	0,670035064	0,92553178	0,836152434	0,894488651
Haemophilus	104,3583908	(-180.4733456;389.1901341)	0,473788559	0,804050712	0,944463611	0,982653769
NA	28,48190498	(-129.4416489;186.405459)	0,723813236	0,951297396	0,96129173	0,982653769
Enterococcaceae_unclassified	1,035811543	(-76.81072792;78.88235098)	0,979194403	0,979194403	0,987627447	0,987627447

Estimates from mixed effect linear regressions with random interce

*p value is derived by (LR) Likelihood Ratio test

Supplementary Table 6B: Summary statistics for associations of genus abundances salivary microbiome) with glucose time series and interaction effects with time

Genus	b (main effect)	95%- Confidence interval	p-value (main effect)	FDR (main effect)	p-value (interaction effect)	FDR (interaction effect)
NA	71,66902924	(6.408558027;136.9294968)	0,037026629	0,907152407	0,003203504	0,15697172
Pseudomonas	315,2163086	(-197.4128423;827.8454791)	0,232153997	0,986654103	0,018567957	0,454914946
Oribacterium	51,47757721	(-91.17151119;194.1266656)	0,480826348	0,986654103	0,033440206	0,546190035
Rothia	-62,11489487	(-110.5862569;-13.64353014)	0,016404819	0,803836118	0,062748052	0,768663639
Pasteurellaceae_unclassified	-538,755127	(-1184.34207;106.8318192)	0,107558049	0,966548034	0,086413838	0,846855612
Corynebacterium	-235,8709259	(-481.9118511;10.1699853)	0,065987922	0,966548034	0,111358881	0,909430861
Actinomyces	-8,799267769	(-126.5086069;108.9100721)	0,883514643	0,986654103	0,140803605	0,984926943
Caulobacter	858,7908936	(-1236.327924;2953.909712)	0,423252881	0,986654103	0,163654223	0,984926943
Neisseriaceae_unclassified	-252,427536	(-661.2513023;156.3962207)	0,230732068	0,986654103	0,225867182	0,984926943
Lachnospiraceae_unclassified	-33,0063858	(-768.4632595;702.4504911)	0,929915905	0,986654103	0,228755012	0,984926943
Actinobacillus	18,01016235	(-37.28829793;73.3086216)	0,524277687	0,986654103	0,239835948	0,984926943
Planococcaceae_unclassified	305,7386169	(-76.54722157;688.0244745)	0,122775838	0,966548034	0,264271349	0,984926943
Catonella	1672,73999	(-717.4814824;4062.961495)	0,174738526	0,966548034	0,28511104	0,984926943
Prevotella	-422,5634155	(-925.5687579;80.44190248)	0,104540363	0,966548034	0,285966158	0,984926943
Prevotellaceae_ge	1358,676758	(-2799.42446;5516.778053)	0,523086131	0,986654103	0,356336921	0,984926943
Streptococcus	-1,438591003	(-37.86252787;34.9853458)	0,938296258	0,986654103	0,359051377	0,984926943
Absconditabacteriales_(SR1)_ge	202,8632355	(-286.1486511;691.8751221)	0,417999297	0,986654103	0,400627315	0,984926943
Lautropia	-56,532444	(-187.2940235;74.2291364)	0,398667902	0,986654103	0,405737996	0,984926943
Streptobacillus	-107,3904495	(-287.7188109;72.93791096)	0,247097194	0,986654103	0,434587151	0,984926943
Atopobium	-144,511795	(-1143.487549;854.463966)	0,776800036	0,986654103	0,460565001	0,984926943
Veillonella	-1,693063974	(-120.3030079;116.9168801)	0,977679372	0,986654103	0,462737709	0,984926943
Fluviicola	1022,150513	(-1997.464575;4041.765548)	0,508067429	0,986654103	0,485102206	0,984926943
Cardiobacterium	-586,0582886	(-1638.01339;465.8967914)	0,278615177	0,986654103	0,516401589	0,984926943
Micrococcaceae_unclassified	6,391338825	(-36.05230672;48.83498405)	0,768023849	0,986654103	0,522024572	0,984926943
Prevotellaceae_unclassified	-383,3343811	(-1503.385799;736.717037)	0,503713191	0,986654103	0,526878178	0,984926943
Oxalobacteraceae_unclassified	323,188385	(-845.6025831;1491.979348)	0,588548243	0,986654103	0,5461936	0,984926943
Campylobacter	-534,687439	(-1583.475195;514.1002574)	0,321311712	0,986654103	0,564714849	0,984926943
Enterococcaceae_unclassified	-23,49117851	(-900.0378429;853.0554876)	0,958111167	0,986654103	0,586419106	0,984926943
Porphyromonas	-55,04497147	(-829.2721954;719.1822489)	0,889197767	0,986654103	0,594605148	0,984926943
Acinetobacter	3,694732666	(-24.50411871;31.89358408)	0,797410071	0,986654103	0,603016496	0,984926943
Capnocytophaga	-228,5159149	(-539.2199875;82.18815076)	0,154702768	0,966548034	0,657697856	0,998857319
Neisseria	7,498488426	(-90.01823052;105.0152075)	0,88020736	0,986654103	0,760572076	0,998857319
Alloprevotella	-23,61768723	(-128.0074548;80.77208013)	0,657918632	0,986654103	0,780678213	0,998857319
Paucibacter	37,53504562	(-262.1047986;337.1748905)	0,806101143	0,986654103	0,816004694	0,998857319
Comamonadaceae_unclassified	-33,73295212	(-392.0795351;324.6136296)	0,853665471	0,986654103	0,837947011	0,998857319
Selenomonas	39,64492035	(-191.6480587;270.9379002)	0,736990154	0,986654103	0,846077502	0,998857319
Flavobacterium	185,7135315	(-265.3849365;636.8119863)	0,421415359	0,986654103	0,854834676	0,998857319
Weeksellaceae_unclassified	-840,2153931	(-2046.729217;366.2984407)	0,177529231	0,966548034	0,857837081	0,998857319
Haemophilus	-82,1760025	(-738.6466715;574.2946691)	0,806323528	0,986654103	0,892315507	0,998857319
Bradyrhizobium	-5,568161964	(-657.9837782;646.8474544)	0,986654103	0,986654103	0,932013929	0,998857319
Afipia	-9,363845825	(-1075.393483;1056.665791)	0,986264408	0,986654103	0,936608195	0,998857319
Fusobacterium	64,81038666	(-105.9476406;235.5684187)	0,458085388	0,986654103	0,942427456	0,998857319
Methylobacterium-Methylorubrum	-129,619278	(-742.4191274;483.1805685)	0,678818047	0,986654103	0,952181458	0,998857319
Sphingomonas	89,07470703	(-258.7685058;436.9179241)	0,616137981	0,986654103	0,953371525	0,998857319
Xanthobacteraceae_unclassified	1224,382446	(-10338.20279;12786.96758)	0,835632205	0,986654103	0,957817376	0,998857319
Alysiella	174,8852386	(-622.8142673;972.584753)	0,667764604	0,986654103	0,963723123	0,998857319
Comamonas	157,7075195	(-227.2251641;542.6402184)	0,423884004	0,986654103	0,98772186	0,998857319
Lachnoanaerobaculum	31,82322121	(-590.4975114;654.1439528)	0,920173168	0,986654103	0,996403217	0,998857319
Aquabacterium	-41,49831009	(-347.4875583;264.4909392)	0,790482938	0,986654103	0,998857319	0,998857319

Estimates from mixed effect linear regressions with random intercepts for the individual; age, sex, BMI, and study group included as covariates; p-value for interaction terms derived from likelihood ratio tests.
*p value is derived by (LR) Likelihood Ratio test

Supplementary Table 7A: Summary statistics for associations of genus abundances (buccal microbiome) with cortisol time series and interaction effects with time

Genus	prevalence	b_coeff (main effect)	95%- Confidence interval	p-value (main effect)	p-value (interaction effect)	FDR (interaction effect)
Planococcaceae_unclassified	0,76	-17,30	(-32.89125518;-1.705758407)	0,03	0,24	1,00
Streptobacillus	0,99	1,02	(.0401121602;2.005533621)	0,04	0,34	1,00
Veillonella	1,00	0,28	(-.035300017;.5946412999)	0,08	0,09	1,00
Alloprevotella	0,87	-1,18	(-2.550843655;.1937288759)	0,10	0,90	1,00
Fusobacterium	1,00	1,09	(-.3057351402;2.476856314)	0,13	0,38	1,00
Veillonellaceae_unclassified	0,82	-2,67	(-6.190802511;.8520773856)	0,14	0,76	1,00
Rothia	1,00	-0,27	(-.6302254698;.0908493595)	0,15	0,99	1,00
Neisseriaceae_unclassified	0,87	3,22	(-1.21420032;7.656647285)	0,16	0,87	1,00
Tannerella	0,66	7,83	(-4.464000666;20.12563489)	0,21	0,89	1,00
Oxalobacteraceae_unclassified	0,56	-8,60	(-22.219216;5.009876934)	0,22	0,99	1,00
Pasteurellaceae_unclassified	0,87	2,72	(-2.107110326;7.538592372)	0,27	0,32	1,00
Capnocytophaga	0,90	2,68	(-2.176065162;7.526155985)	0,28	0,93	1,00
Methylobacterium-Methylorubrum	0,72	-5,22	(-15.25333808;4.80373884)	0,31	0,89	1,00
Neisseria	1,00	-0,31	(-.9117280611;.2891717398)	0,31	0,99	1,00
Flavobacterium	0,86	-2,69	(-7.908054773;2.521298905)	0,31	1,00	1,00
Bradyrhizobium	0,65	-6,17	(-19.10623633;6.774087124)	0,35	0,76	1,00
Comamonadaceae_unclassified	0,79	-3,12	(-9.999998211;3.768656846)	0,38	0,99	1,00
Treponema	0,56	-4,44	(-14.53644103;5.658813853)	0,39	0,53	1,00
Cardiobacterium	0,70	2,68	(-3.49684255;8.858701976)	0,40	0,69	1,00
Unclassified	1,00	0,35	(-.4685915171;1.164145752)	0,40	0,23	1,00
Paucibacter	0,72	-1,58	(-6.300603234;3.148534025)	0,51	0,70	1,00
Micrococcaceae_unclassified	1,00	-0,08	(-.3283819815;.1757413291)	0,55	0,92	1,00
Lachnoanaerobaculum	0,96	1,07	(-2.513225791;4.648669947)	0,56	0,99	1,00
Actinomyces	1,00	0,07	(-.1780194854;.3251217916)	0,57	0,02	1,00
Prevotella	0,96	0,83	(-2.070380038;3.72458621)	0,58	0,97	1,00
Porphyromonas	0,97	0,59	(-1.897204986;3.068367391)	0,64	0,20	1,00
Lachnospiraceae_unclassified	0,94	0,99	(-3.234350716;5.206497489)	0,65	0,82	1,00
Actinobacillus	1,00	-0,05	(-.2723950848;.1753788743)	0,67	0,95	1,00
Terrisporobacter	0,55	-4,06	(-23.50704122;15.38925902)	0,68	1,00	1,00
Enterococcaceae_unclassified	0,92	-0,13	(-.8055423947;.5387257108)	0,70	1,00	1,00
Haemophilus	0,93	0,38	(-1.657616495;2.425957512)	0,71	0,99	1,00
Lautropia	0,93	0,31	(-1.458950805;2.075481989)	0,73	0,20	1,00
Campylobacter	0,99	1,20	(-5.823102524;8.220097917)	0,74	1,00	1,00
Sphingomonas	0,75	-0,87	(-6.137447971;4.38758299)	0,74	0,64	1,00
Prevotellaceae_unclassified	0,89	-0,59	(-4.156007809;2.973056018)	0,75	0,60	1,00
Atopobium	0,77	-2,20	(-15.67222224;11.2667349)	0,75	0,22	1,00
Selenomonas	0,83	0,52	(-3.833187854;4.883022041)	0,81	0,99	1,00
Oribacterium	0,94	0,29	(-2.53879131;3.11511402)	0,84	0,34	1,00
Catonella	0,65	4,90	(-50.18220002;59.98832539)	0,86	0,85	1,00
Weeksellaceae_unclassified	0,90	0,64	(-14.31087392;15.59541944)	0,93	0,91	1,00
Acinetobacter	0,72	-0,21	(-5.218162647;4.790753477)	0,93	0,18	1,00
Corynebacterium	0,79	0,10	(-2.52907703;2.725147025)	0,94	0,85	1,00
Streptococcus	1,00	0,00	(-.1121705326;.1043571584)	0,94	0,90	1,00
Alysiella	0,82	0,19	(-4.969344457;5.339370519)	0,94	0,93	1,00
Parvimonas	0,79	-0,02	(-2.510027894;2.463321055)	0,99	0,95	1,00
Prevotellaceae_ge	0,80	0,12	(-26.28759593;26.52820717)	0,99	0,94	1,00

Estimates derived from linear regressions adjusted for age, sex, BMI, study group

*p value is derived by (LR) Likelihood Ratio test

Supplementary Table 7B: Summary statistics for associations of genus abundances (salivary microbiome) with cortisol time series and interaction effects with time

Genus	prevalence	b_coeff (main effect)	95%- Confidence interval	p-value (main effect)	p-value (interaction effect)	FDR (interaction effect)
Acinetobacter	1,00	0,18	(-.0173579653;.3680916249)	0,08	4,64E-03	0,23
Haemophilus	0,55	2,61	(-1.025110159;6.244048434)	0,16	0,01	0,27
Actinomyces	1,00	-0,04	(-.6737833526;.5997424933)	0,91	0,05	0,62
Absconditabacteriales_(SR1)_ge	0,58	4,39	(.2873079538;8.493951631)	0,04	0,05	0,62
Campylobacter	0,75	-1,52	(-8.767040396;5.726993083)	0,68	0,14	1,00
Catonella	0,72	6,39	(-8.227744859;21.01379529)	0,39	0,19	1,00
Pseudomonas	0,77	0,60	(-1.172486193;2.365594936)	0,51	0,21	1,00
Corynebacterium	0,94	-0,66	(-2.289063649;.9777482375)	0,43	0,28	1,00
Rothia	1,00	-0,02	(-.4159652055;.3666929087)	0,90	0,56	1,00
Sphingomonas	0,99	0,46	(-1.152196258;2.066625705)	0,58	0,60	1,00
Weeksellaceae_unclassified	0,96	-2,50	(-7.770870799;2.772023768)	0,35	0,62	1,00
Afipia	0,87	6,07	(-.9842391396;13.1336487)	0,09	0,64	1,00
Lachnospiraceae_unclassified	0,79	0,83	(-1.3443422;3.005451101)	0,46	0,69	1,00
Fusobacterium	0,96	-0,23	(-1.396944456;.9352799676)	0,70	0,69	1,00
Flavobacterium	0,99	0,41	(-2.097197455;2.919415027)	0,75	0,69	1,00
Prevotella	0,82	-2,29	(-5.71407377;1.131168335)	0,19	0,73	1,00
Neisseriaceae_unclassified	0,80	-0,84	(-4.175668566;2.5033274)	0,62	0,75	1,00
Streptococcus	1,00	-0,07	(-.2811727615;.1416300235)	0,52	0,78	1,00
Fluviicola	0,54	-15,92	(-38.72070863;6.884472994)	0,17	0,79	1,00
Streptobacillus	0,90	-0,25	(-1.430689891;.9300719586)	0,68	0,84	1,00
Xanthobacteraceae_unclassified	0,51	10,26	(-80.88715023;101.4151037)	0,83	0,86	1,00
Cardiobacterium	0,73	-0,67	(-5.649386126;4.310240381)	0,79	0,89	1,00
Lautropia	0,83	-0,24	(-1.235972983;.7603160413)	0,64	0,89	1,00
Comamonadaceae_unclassified	1,00	0,74	(-1.312375893;2.790637429)	0,48	0,89	1,00
Capnocytophaga	0,82	-0,95	(-3.504517168;1.602214683)	0,47	0,90	1,00
Atopobium	0,58	1,71	(-6.194867787;9.622876281)	0,67	0,90	1,00
Aquabacterium	0,94	-0,30	(-2.242250031;1.635562063)	0,76	0,92	1,00
Bradyrhizobium	1,00	1,46	(-2.464799756;5.377468547)	0,47	0,92	1,00
Unclassified	1,00	0,20	(-.2690682783;.6627175611)	0,41	0,94	1,00
Alysiella	0,56	1,97	(-4.2073162;8.142176445)	0,53	0,94	1,00
Veillonella	0,96	-0,55	(-1.226354703;.1326241322)	0,12	0,95	1,00
Enterococcaceae_unclassified	0,66	1,70	(-5.057470942;8.461145753)	0,62	0,96	1,00
Porphyromonas	0,83	0,49	(-4.534820794;5.515739173)	0,85	0,97	1,00
Prevotellaceae_unclassified	0,80	-2,08	(-8.358541479;4.189907245)	0,52	0,98	1,00
Neisseria	0,96	-0,63	(-1.392344594;.1284371906)	0,11	0,98	1,00
Oribacterium	0,89	0,23	(-.5329481782;1.002510421)	0,55	0,98	1,00
Caulobacter	0,68	-1,67	(-7.251899208;3.904464158)	0,56	0,99	1,00
Paucibacter	1,00	0,31	(-1.066647124;1.679968281)	0,66	0,99	1,00
Selenomonas	0,83	0,01	(-1.177934668;1.193165494)	0,99	0,99	1,00
Comamonas	1,00	0,61	(-1.47137521;2.700871989)	0,56	0,99	1,00
Prevotellaceae_ge	0,66	-4,06	(-17.74149045;9.626239586)	0,56	0,99	1,00
Planococcaceae_unclassified	0,96	-0,35	(-2.294953647;1.591896166)	0,72	1,00	1,00
Alloprevotella	0,76	0,15	(-.7625313923;1.059579445)	0,75	1,00	1,00
Micrococcaceae_unclassified	1,00	-0,12	(-.4208237594;.1869974647)	0,45	1,00	1,00
Pasteurellaceae_unclassified	0,80	-1,42	(-5.507462055;2.66381551)	0,50	1,00	1,00
Oxalobacteraceae_unclassified	0,92	3,05	(-3.245567457;9.346102982)	0,34	1,00	1,00
Methylobacterium-Methylorubrum	0,99	0,25	(-3.061520674;3.558241765)	0,88	1,00	1,00
Actinobacillus	0,94	-0,17	(-.5032040193;.1632083274)	0,32	1,00	1,00
Lachnoanaerobaculum	0,66	0,60	(-4.581815729;5.7751153)	0,82	1,00	1,00

Estimates derived from linear regressions adjusted for age, sex, BMI, study group

*p value is derived by (LR) Likelihood Ratio test

Supplementary Table8A: Summary statistics for associations of diversity indices plasma-glucose kinetics

		Diversity Index	b_coeff (main effect)	95%- Confidence interval	p-value (main effect)	p-value (interaction effect)
Salivary microbiome	Time series	Inverse Simpson Diversity Index	-0,074	(-.6642193198;.5154210925)	8,05E-01	2,36E-01
		Shannon Evenness Index	5,976	(-50.50314081;62.45518)	8,36E-01	2,09E-01
	Baseline	Inverse Simpson Diversity Index	-	(-.675673306;.675673306)	0,233	-
		Shannon Evenness Index	-	(-64.46648912;64.46648912)	0,220	-
Buccal microbiome	Time series	**Inverse Simpson Diversity Index**	**-0,066**	**(-.4586122105;.326646541)**	**7,42E-01**	**1,68E-02**
		**Shannon Evenness Index**	**-8,933**	**(-48.56170781;30.69512036)**	**6,59E-01**	**4,47E-04**
	Baseline	Inverse Simpson Diversity Index	-	(-.436742872;.436742872)	0,494	-
		Shannon Evenness Index	-	(-44.45878933;44.45878933)	0,873	-

Estimates derived from linear regressions adjusted for age, sex, BMI, study group

*p value is derived by (LR) Likelihood Ratio test

Supplementary Table8B: Summary statistics for associations of diversity indices with salivary-cortisol kinetics

		Diversity Index	b_coeff (main effect)	95%- Confidence interval	p-value (main effect)	p-value (interaction effect)
Salivary microbiome	Time series	Inverse Simpson Diversity Index	0,000	(-.0041569427;.0033395058)	0,831	1,93E-01
		**Shannon Evenness Index**	**0,020**	**(-.3856426047;.4247673948)**	**0,925**	**3,33E-03**
	Baseline	Inverse Simpson Diversity Index	-	(.0820735533;.0887378383)	0,532	-
		Shannon Evenness Index	-	(-.2721876208;.4408103139)	0,174	-
Buccal microbiome	Time series	**Inverse Simpson Diversity Index**	**0,002581816**	**(.0001850768;.0049785555)**	**3,76E-02**	**1,19E-02**
		Shannon Evenness Index	0,070689768	(-.1786407203;.3200202514)	5,79E-01	8,41E-01
	Baseline	Inverse Simpson Diversity Index	-	(.0820735533;.0887378383)	0,532	-
		Shannon Evenness Index	-	(-.2721876208;.4408103139)	0,174	-

Estimates derived from linear regressions adjusted for age, sex, BMI, study group

*p value is derived by (LR) Likelihood Ratio test
